# CircNOLC1 Promotes Colorectal Cancer Liver Metastasis by Interacting with AZGP1 and Sponging miR‐212‐5p to Regulate Reprogramming of the Oxidative Pentose Phosphate Pathway

**DOI:** 10.1002/advs.202205229

**Published:** 2023-10-23

**Authors:** Menglang Yuan, Xinsheng Zhang, Fangxia Yue, Feifan Zhang, Sufen Jiang, Xu Zhou, Jinjuan Lv, Zhenyu Zhang, Yuzhu Sun, Zihao Chen, Han Wu, Xiaoqian Liu, Xiaoqi Yu, Bowen Wei, Kexin Jiang, Fang Lin, Yunfei Zuo, Shuangyi Ren

**Affiliations:** ^1^ Department of General Surgery The Second Hospital of Dalian Medical University 116023 Dalian China; ^2^ Department of Clinical Biochemistry College of Laboratory Diagnostic Medicine Dalian Medical University 116044 Dalian China; ^3^ Department of Oncology Sidney Kimmel Comprehensive Cancer Center School of Medicine Johns Hopkins University Baltimore MD 21287 USA

**Keywords:** AZGP1, circNOLC1, miR‐212‐5p, oxidative pentose phosphate pathway

## Abstract

Liver metastasis is a common cause of death in progressive colorectal cancer patients, but the molecular mechanisms remain unclear. Here, it is reported that a conserved and oxidative pentose phosphate pathway‐associated circular RNA, circNOLC1, plays a crucial role in colorectal cancer liver metastasis. It is found that circNOLC1 silencing reduces the oxidative pentose phosphate pathway‐related intermediate metabolites and elevates NADP^+^/NADPH ratio and intracellular ROS levels, thereby attenuating colorectal cancer cell proliferation, migration, and liver metastasis. circNOLC1 interacting with AZGP1 to activate mTOR/SREBP1 signaling, or sponging miR‐212‐5p to upregulate c‐Met expression, both of which can further induce G6PD to activate oxidative pentose phosphate pathway in colorectal cancer liver metastasis. Moreover, circNOLC1 is regulated by the transcription factor YY1 and specifically stabilized HuR induces its parental gene mRNA expression. The associations between circNOLC1 and these signaling molecules are validated in primary CRC and corresponding liver metastasis tissues. These findings reveal that circNOLC1 interacting with AZGP1 and circNOLC1/miR‐212‐5p/c‐Met axis plays a key role in oxidative pentose phosphate pathway‐mediated colorectal cancer liver metastasis, which may provide a novel target for precision medicine of colorectal cancer.

## Graphical abstract

Liver metastasis is a common cause of death in progressive colorectal cancer patients. This study identifies a novel pathway underlying colorectal cancer liver metastasis by circNOLC1‐mediated oxidative pentose phosphate pathway reprogramming. This effect is achieved by circNOLC1 interacting with AZGP1 and sponging miR‐212‐5p to induce G6PD activity, which can provide a novel target for precision medicine of colorectal cancer.

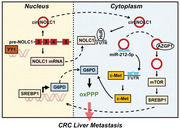

## 1 Introduction

Metastasis is the leading cause of death in patients with colorectal cancer (CRC), and the liver is the most common site for distant metastasis.^[^ ^1^ ^]^ Among them, only about a quarter of patients affected are amenable to resection, the cause that the 5‐year survival rate is less than 10% after liver metastasis diagnosis.^[^ ^2^, ^3^ ^]^ Therefore, uncovering the underlying genetic alterations and regulatory targets in CRC liver metastases is urgently necessary for the development of novel anticancer therapies.

Metabolic reprogramming has emerged as a key hallmark of cancer.^[^ ^4^ ^]^ Growing evidence suggests that metabolic reprogramming is required during metastatic dissemination and colonization in CRC.^[^ ^5^ ^]^ Metabolic reprogramming‐involved multiple metabolic pathways including glucose metabolism, lipid homeostasis, and amino acid metabolism may be involved in CRC liver metastasis.^[^ ^6^, ^7^, ^8^, ^9^ ^]^ The pentose phosphate pathway (PPP), a branch of glucose metabolism, has become the focus of metabolic reprogramming. The oxidative pentose phosphate pathway (oxPPP) can provide both nucleotide precursors for tumor growth and nicotinamide adenine dinucleotide phosphate (NADPH) for tumor metastasis.^[^ ^10^, ^11^ ^]^ Glucose‐6‐phosphate dehydrogenase (G6PD) is the first rate‐limiting enzyme in the oxPPP, and its function is strictly regulated in normal cells but highly activated in cancer cells. Recent reports have shown that G6PD activity may be affected by numerous factors, such as tumor suppressor p53, p73, PTEN, transcription factor YY1, protein kinase mTOR, and Plk1.^[^ ^12^, ^13^, ^14^, ^15^, ^16^, ^17^ ^]^ However, despite the crucial roles of G6PD and the oxPPP in CRC, their regulatory mechanisms in liver metastases still remain unclear.

CircRNAs play an important role in CRC growth and metastasis. By sponging, recruitment, or scaffolding of microRNAs or proteins, and translation of specific peptides, circRNAs can promote or suppress various hallmarks of cancer. It has been reported that *N^6^*‐methyladenosine modification of circNSUN2 modulates cytoplasmic export and promotes epithelial‐to‐mesenchymal transition (EMT) in CRC liver metastasis via forming a circNSUN2/IGF2BP2/HMGA2 RNA‐protein ternary complex.^[^ ^18^ ^]^ CircPPP1R12A encodes a functional protein, circPPP1R12A‐73aa, that facilities the occurrence and liver metastasis of colon cancer by activating the Hippo‐YAP (Yes‐associated protein) signaling pathway.^[^ ^19^ ^]^ In addition, circPPFIA1s functioned as sponges of oncogenic miR‐155‐5p and Hu antigen R (HuR) to inhibit the CRC liver metastasis through the miR‐155‐5p/CDX1 and HuR/RAB36 pathways.^[^ ^20^ ^]^ Recently, it has become evident that multiple metabolic pathways including glycolysis, fatty acid oxidation, and amino acid metabolism in CRC are tightly regulated by circRNAs.^[^ ^21^, ^22^, ^23^ ^]^ Notably, however, little is known about circRNA‐induced metabolic pathways in CRC liver metastasis.

RNA binding proteins (RBPs) play key roles in circRNA‐mediated gene regulation, and obtaining the full interaction map of proteins bound to a circRNA of interest is critical to our understanding of its function.^[^ ^24^ ^]^ It is reported that circSTX6 was confirmed to participate in the ubiquitin‐dependent degradation of hypoxia‐inducible factor 1‐alpha (HIF1A) by interacting with CUL2.^[^ ^25^ ^]^ circMYBL2 was found to enhance the translational efficiency of the FLT3 kinase by increasing the binding of polypyrimidine tract‐binding protein 1 (PTBP1) to FLT3 messenger RNA.^[^ ^26^ ^]^ In addition, circACTN4 promotes intrahepatic cholangiocarcinoma progression by recruiting YBX1 to initiate FZD7 transcription.^[^ ^27^ ^]^ However, to date, potent and sensitive circRNA binding proteins for CRC liver metastasis are mostly unknown.

c‐Met is a hepatocyte growth factor receptor with tyrosine kinase activity that is associated with a variety of oncogene products and regulatory proteins and is involved in the regulation of cellular signal transduction and cytoskeletal rearrangement. It has been reported that c‐Met is a key factor in promoting liver metastasis of colorectal cancer. Upregulation of c‐Met expression in colon cancer cells with overexpression of MACC1 (metastasis‐associated in colon cancer‐1) significantly increased the liver metastasis rate.^[^ ^28^ ^]^ Sprouty‐2 was found to increase the metastatic potential by regulating the expression of c‐Met in colon cancer cells.^[^ ^29^ ^]^ Our previous study found that liver and lymph node sinus endothelial cell c‐type lectin (LSECtin) promoted liver metastasis by upregulating c‐Met expression in colon cancer cells.^[^ ^30^ ^]^

Here, we identified the mechanism by which circNOLC1 modulates oxPPP‐mediated metabolic reprogramming in human CRC liver metastasis. We discovered that circNOLC1 interacted with AZGP1 to activate mTOR/SREBP1 signaling, or sponged miR‐212‐5p to upregulate c‐Met expression, both of which could further induce G6PD to activate oxPPP in CRC liver metastasis. We also found that circNOLC1 was transcriptionally regulated by YY1 and specifically stabilized HuR‐induced parental gene mRNA expression. The identification of the oxPPP regulated by circNOLC1 interacted with AZGP1 and sponged miR‐212‐5p will help further our understanding of how metabolic reprogramming elicits CRC liver metastasis.

## 2 Results

### 2.1 Identification and Characterization of circNOLC1, a circRNA that Specifically Showed a Higher Expression Level in Metastatic CRC

**Figure 1:**
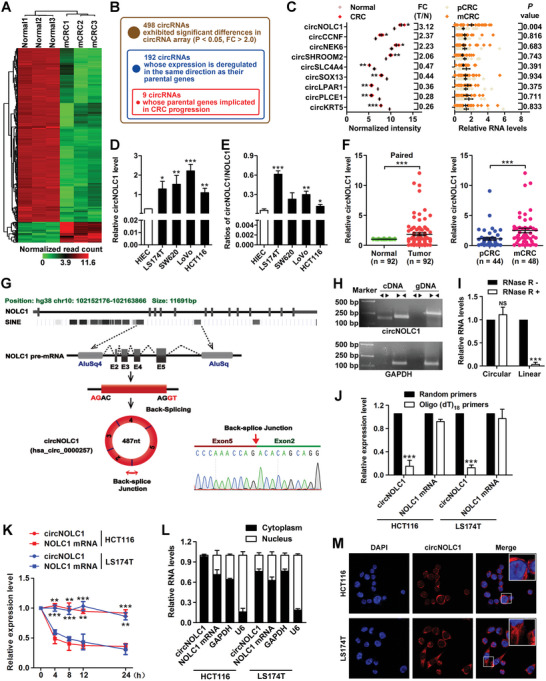
Identification and characterization of circNOLC1, a circRNA that specifically showed a higher expression level in metastatic CRC. A) Clustered heatmap of the differentially expressed circRNAs in three paired metastatic CRC and adjacent normal tissues. B) Identification of circRNAs having significant expression differences in malignant CRC samples compared with normal colon samples. C) Top 9 circRNAs significantly upregulated or downregulated in three paired CRC and adjacent normal tissues. D) CircNOLC1 expression in CRC cell lines was higher than that in the normal intestinal epithelial cell line as determined by qPCR. E) Comparison of the ratio of circular to linear product reads between normal and CRC cell lines. F) Differential expression of circNOLC1 between 92 paired normal colon and primary CRC (left) and primary CRC and liver metastasis samples (right) as determined by qPCR. G) Structures of the NOLC1 genomic DNA and transcript. circNOLC1 is produced from exons 2–5 of NOLC1 by back‐splicing. Sanger sequencing identified the back‐splice junction of circNOLC1. H) Divergent and convergent primers amplify circNOLC1 in cDNA and genomic DNA of HCT116. I) The resistance of circular and linear NOLC1 to RNase R digestion. J) Random or oligo(dT)18 primers were used for reverse transcription. The relative RNA levels were determined by qPCR and normalized to the values using random primers. K) circNOLC1 and NOLC1 levels in HCT116 and LS174T cells were determined by qPCR after actinomycin D treatment for 4, 8, 12, and 24 h. L) Identification of circNOLC1 and NOLC1 mRNA distribution by qPCR analysis. GAPDH and U6 were used as the cytoplasmic and nuclear markers, respectively. M) RNA FISH for circNOLC1. Cy3 dye and DAPI stain. ^*^ *p* < 0.05, ^**^ *p* < 0.01, ^***^ *p* < 0.001. DAPI, 4′,6‐diamidino‐2‐phenylindole; FISH, fluorescence in situ hybridization; CRC, colorectal cancer; qPCR, quantitative reverse transcription PCR.

To discover circRNAs specifically functioning in metastatic CRC, we compared three pairs of metastatic CRC tissues and their matched nontumor tissues by using a circRNA microarray. The volcano plot shows the variation in circRNA expression between the CRC tissues and paired adjacent normal tissues (Figure S1A, Supporting Information). A total of 498 circRNAs in the microarray exhibited significant differences (P < 0.05 and fold change > 2.0), 53 were upregulated and 445 were downregulated (**Figure** **1**A). Chromosome analysis showed all chromosomes produced abnormally expressed circRNAs (Figure S1B, Supporting Information). Among the 498 differentially expressed circRNAs, 449 (90.16%) were from exons (Figure S1C, Supporting Information), 192 of these circRNAs whose expression was deregulated in the same direction as their parental genes obtained from The Cancer Genome Atlas (TCGA). We cross‐referenced the corresponding parental genes against the 192 circRNAs, deriving a prioritized list of 27 parental genes with differential expression (Table S1, Supporting Information). Among them, 9 circRNAs whose parental genes were previously reported to be associated with CRC progression (Figure 1B; Table S2, Supporting Information).

Of note, circNOLC1 (circBase ID: hsa_circ_0000257) and its parental gene NOLC1, respectively, were one of the most abundant differentially expressed transcripts according to their normalized read counts in our microarray and TPM values in the TCGA‐COADREAD cohort (Figure 1C; Figure S1D, Supporting Information). Moreover, circNOLC1 was the most differential between CRC with metastasis and without metastasis (Figure 1C). Consistently, circNOLC1 was highly abundant in both normal human intestinal epithelial and CRC cell lines (Figure 1D). Furthermore, the back‐spliced ratio of circNOLC1 was more highly expressed than its linear isoforms (Figure 1E). We next measured circNOLC1 expression in 92 human CRC and corresponding normal tissues by qPCR. As expected, circNOLC1 was significantly different between CRC and normal tissues and was higher in CRCs with metastasis than without metastasis (Figure 1F). Therefore, we chose circNOLC1 for further study.

According to the human reference genome (GRCh38/hg38), we identify that circNOLC1 is 487 bp in spliced length and consists of 4 exons (exons 2–5) from the NOLC1 gene. Back‐ splicing in the RT‐PCR product of circNOLC1 with the expected size was confirmed by Sanger sequencing (Figure 1G). To further confirm the circular characteristics of circNOLC1, RNA and genomic DNA were isolated from CRC‐derived cells. We amplified circNOLC1 and NOLC1 from cDNA and genomic DNA utilizing divergent and convergent primers and gel electrophoresis showed that circNOLC1 only exists in the cDNA (Figure 1H). Furthermore, we digested total RNA with RNase R exonuclease and found that circNOLC1, but not linear NOLC1, could be amplified after RNase R digestion (Figure 1I). With the oligo(dT)_18_ primers compared with the random primers, the relative expression of circNOLC1 was significantly decreased, while NOLC1 mRNA was not (Figure 1J). Moreover, we used actinomycin D to inhibit transcription and found that circNOLC1 was more stable than NOLC1 mRNA (Figure 1K). qPCR and fluorescence in situ hybridization (FISH) for circNOLC1 showed the predominant cytoplasmic distribution of circNOLC1 (Figure 1L,M). Finally, we excluded the possibility that circNOLC1 is translated as occurs with some circRNAs through the open reading frame (ORF) and internal ribosome entry site (IRES).^[^ ^32^ ^]^ Using CPC2,^[^ ^33^ ^]^ we predicted that circNOLC1 has no protein‐coding potential (Table S3, Supporting Information). Together, these results suggest that circNOLC1 is an abundant, circular, and stable transcript, which may potentially contribute to CRC metastasis.

### 2.2 circNOLC1 Silencing Specifically Impairs the Growth and Liver Metastasis of CRC Cells

**Figure 2:**
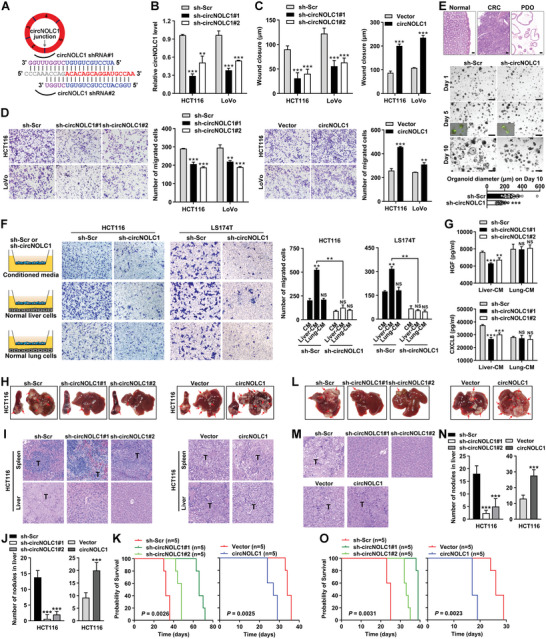
circNOLC1 silencing specifically impairs the growth and liver metastasis of CRC cells. A) Schematic of the shRNAs used for targeting circNOLC1 at the junction sequences. The blue and purple sequences show the corresponding 5′ and 3′ exon sequences forming the junction. B) Efficient knockdown of circNOLC1 in HCT116 and LS174T cells was detected by qPCR. C,D) The effect of circNOLC1 knockdown or overexpression on the migration ability of human CRC cells was evaluated by wound healing (C) and Transwell (D) assays. E) Representative images of H&E staining both CRC patient‐derived organoids (PDO) and their matching patient tissue (top). Matrigel‐suspended organoids transduced with lentiviral vectors containing circNOLC1 knockdown cultured for 1, 5, and 10 days (bottom). Organoid diameter on day 10. Scale bar: 500 µm. F) Schematic for the coculture experiment. The effect of circNOLC1 knockdown on migration with liver or lung organotropism of CRC cells was evaluated by the Boyden chamber coculture system (left). Quantitation of the indicated cells counted individually per visual field (right). G) HGF and CXCL8 levels in the supernatant co‐cultured circNOLC1‐depleted HCT116 cells with normal hepatocyte L‐02 (Liver‐CM) or lung epithelial BEAS‐2B cells (Lung‐CM). H) The spleen and liver were examined at the gross anatomical level for tumors and metastases after intra‐splenic injection with circNOLC1 knockdown or overexpressed HCT116 cells. The red arrows indicate tumor foci. I) Representative H&E staining showing primary (upper) and metastatic (lower) nodules. T, tumor. J) The number of metastatic nodules in the livers. K) Kaplan–Meier survival curves for mice implanted with circNOLC1 knockdown or overexpressed HCT116 cells. L) The liver was examined at the gross anatomical level for tumors and metastases after liver capsule (Glisson's capsule) injection with circNOLC1 knockdown or overexpressed HCT116 cells. The red arrows indicate tumor foci. M) Representative H&E staining showing metastatic nodules. T, tumor. N) The number of metastatic nodules in the livers. O) Kaplan–Meier survival curves for mice implanted with circNOLC1 knockdown or overexpressed HCT116 cells. *p* values were calculated using the log‐rank (Mantel–Cox) test. ^**^ *p* < 0.01, ^***^ *p* < 0.001, NS, not significant. H&E, hematoxylin and eosin; shRNA, short hairpin RNA; CRC, colorectal cancer; qPCR, quantitative reverse transcription PCR.

To evaluate the biological roles of circNOLC1 in greater detail, two short hairpin RNAs (shRNAs) spanning the back‐splicing junction of circNOLC1 were designed to silence circNOLC1 expression via lentiviral transduction (**Figure** **2**A; Figure S2A, Supporting Information). We found that the depletion of circNOLC1 efficiently decreased its mRNA level in HCT116 and LoVo cells (Figure 2B). The Cell Counting Kit‐8 (CCK‐8) and colony formation assays revealed a significant delay in the growth of CRC cells after the knockdown of circNOLC1 compared with the negative control (Figure S2B,C,E, Supporting Information). Furthermore, wound healing and Transwell migration assays demonstrated that circNOLC1 silencing markedly decreased the migratory potential of CRC cells (Figure 2C,D). A similar growth and migration inhibitory effect was observed in LS174T circNOLC1‐depleted cells (Figure S2E, Supporting Information). CRC patient‐derived organoids (PDO) growth assay further showed that circNOLC1 had no significant impact on the aggregation of organoids, but circNOLC1 knockdown inhibited organoid growth (Figure 2E). A similar cell growth promotive effect of circNOLC1 overexpression was observed in organoid models (Figure S2F, Supporting Information). To determine whether circNOLC1 mediates CRC metastasis to specific organs, we used a Boyden chamber coculture system and found that culture media from normal liver cells markedly promoted the metastasis of CRC cells, whereas culture medium from normal lung cells had no effect. This liver organotropism was attenuated in circNOLC1‐depleted cells (Figure 2F). HGF and CXCL8 levels in the supernatant were significantly decreased in circNOLC1‐depleted HCT116 cells only when HCT116 cells were co‐cultured with the normal liver cells, while no changes were observed when co‐cultured with normal lung cells (Figure 2G), suggesting that circNOLC1 was indispensable for CRC liver metastasis. In addition, stable CRC cells expressing negative control or circNOLC1 shRNA were injected into the distal tip of the spleen of nude mice. Seven weeks later, the spleens and livers were removed and embedded in paraffin. We noted that all mice injected with stable HCT116 and LoVo cells expressing control shRNA formed tumors in the spleen and liver metastases; in contrast, few of the mice injected with stable HCT116 and LoVo cells expressing circNOLC1 shRNA formed liver metastases (Figure 2H; Figure S2G, Supporting Information). Moreover, the number of metastatic nodules in the livers was significantly reduced in mice inoculated with CRC cells transduced with circNOLC1 shRNA compared with control (Figure 2I,J). Survival analysis revealed that the survival rate of mice was significantly increased in circNOLC1‐depleted cells (Figure 2K; Figure S2G, Supporting Information). By using the lentiviral overexpression vector, we successfully obtained stable circNOLC1 overexpression in HCT116 and LoVo cells (Figure S2A, Supporting Information). For functional studies, we found that exogenous overexpression of circNOLC1 in CRC cells promoted malignant proliferation and migration in vitro (Figure 2C,D; Figure S2D, Supporting Information) and increased the formation of liver metastasis of CRC cells in vivo (Figure 2H–K; Figure S2H, Supporting Information).

To confirm the role of circNOLC1 in CRC liver metastases, we also established a liver capsule injection model. We noted that all mice injected with stable cells expressing circNOLC1 shRNA led to a reduction in the incidence of macroscopic liver metastasis (Figure 2L; Figure S2I, Supporting Information). Similar to the intra‐splenic injection model, H&E staining and quantification indicated that the number of metastatic nodules in the liver was reduced after being inoculated with the circNOLC1‐depleted CRC cells and were increased after circNOLC1 overexpression (Figure 2M,N). Survival analysis revealed that the survival rate of mice was significantly increased in circNOLC1‐depleted cells and decreased in circNOLC1‐overexpressed cells (Figure 2O; Figure S2I, Supporting Information), indicating that circNOLC1 silencing limits liver metastasis in the liver orthotopic model. Together, these results suggest that circNOLC1 knockdown can significantly impair the growth and liver metastatic ability of CRC cells in vitro and in vivo.

### 2.3 circNOLC1 Regulates G6PD Level and Activity to Promote Pentose Phosphate Pathway in an mTOR‐Dependent Manner

**Figure 3:**
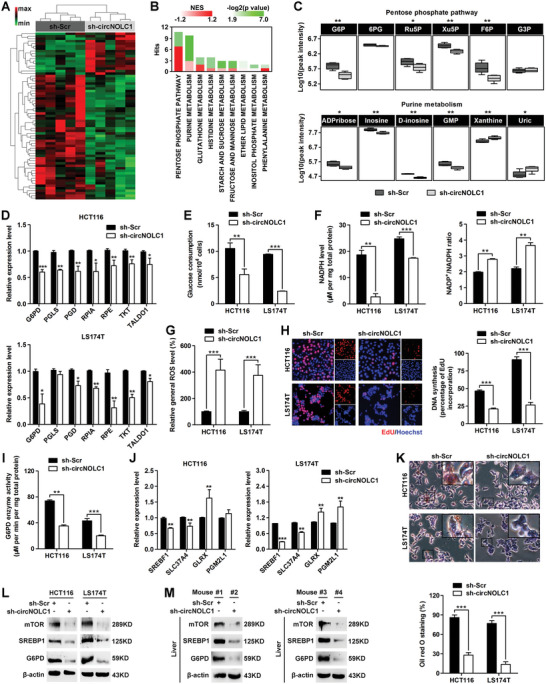
circNOLC1 regulates G6PD level and activity to promote the pentose phosphate pathway in an mTOR‐dependent manner. A) Heatmap of metabolite clusters in HCT116 cells expressing control or circNOLC1 shRNA, as measured by LC–MS/MS‐based metabolomics. B) Integrated pathway analysis of transcriptomic and metabolomic data. The significantly enriched (*q* value < 0.05) genes identified by RNA‐seq and significantly enriched (VIP value ≥1) metabolites identified by metabolomics in circNOLC1‐depleted HCT116 cells compared with control cells were integrated by combining the GSEA and KEGG enrichment analysis results based on gene‐metabolite pathways. The *y*‐axis shows the number of deregulated metabolites and genes in the metabolic pathway. The color bar refers to the enrichment analysis results determining the importance of the genes and metabolites on their position within a metabolic pathway based on the NES and *p*‐value. C) Representation of the downregulated pentose phosphate pathway (upper) and purine metabolism (lower) and MS peak intensity of the corresponding intermediate metabolites. D) The mRNA expression levels of PPP‐related genes in circNOLC1‐silenced CRC cells were analyzed by qPCR. E) Glucose consumption level in circNOLC1‐silenced CRC cells. The total cell number was used for normalization. F) Intracellular NADPH level (left) and NADP+/NADPH ratio (right) in circNOLC1‐silenced CRC cells. G) Intracellular ROS level in circNOLC1‐silenced CRC cells. H) De novo DNA synthesis was evaluated using an EdU incorporation assay. Alexa Fluor 555 dye and Hoechst 33342 stain. I) G6PD activity in circNOLC1‐silenced CRC cells. J) The mRNA expression levels were detected using qPCR after integrated analysis of RNA‐seq profile and the key genes involved in the regulation of the PPP. K) Lipid accumulation in circNOLC1‐silenced CRC cells was stained using Oil red O. Representative images (upper) and quantitative results (lower) are shown. L) The protein levels of mTOR, SREBP1, and G6PD were detected by western blot analysis. M) Stable HCT116 cells expressing control or circNOLC1 shRNA were injected into the spleen. Liver tissue lysates were applied to western blot analysis. ^*^ *p* < 0.05, ^**^ *p* < 0.01, ^***^ *p* < 0.001. NES, normalized enrichment score; GSEA, gene set enrichment analysis; PPP, pentose phosphate pathway; ROS, reactive oxygen species; EdU, 5‐ethynyl‐2′‐deoxyuridine; RNA‐seq, RNA sequencing; CRC, colorectal cancer; qPCR, quantitative reverse transcription PCR.

circNOLC1 parental gene NOLC1 is reported to have GTPase/ATPase activities and participate in the metabolism of small nucleolar RNA.^[^ ^34^ ^]^ Therefore, we assumed that circNOLC1 may promote CRC liver metastasis by mediating metabolic reprogramming. We performed liquid chromatography coupled with tandem mass spectrometry (LC‐MS/MS) and RNA‐sequencing to investigate the metabolic alterations induced by circNOLC1 knockdown. LC‐MS/MS analysis showed the presence of distinct metabolite clusters in circNOLC1‐depleted HCT116 cells compared with control (**Figure** **3**A). Similarity matrix analysis of metabolites suggested that certain metabolites affected by circNOLC1 knockdown were consistent with those in the GEO: GSE41568 colon cancer liver metastasis dataset (Figure S3A, Supporting Information). RNA‐sequencing analysis was then performed to measure the expression levels of the involved metabolic enzymes. Unsupervised hierarchical clustering revealed a total of 152 upregulated genes and 74 downregulated genes in circNOLC1‐depleted HCT116 cells (Figure S3B, Supporting Information). GO analysis revealed that the metabolic process was involved in the most leading affected biological process in circNOLC1‐depleted cells (Figure S3C, Supporting Information).

We next combined metabolomics and transcriptomics to identify metabolic pathways that were likely altered in circNOLC1‐depleted cells compared with control cells. Integrated analysis showed that circNOLC1 knockdown primarily affected the carbohydrate and nucleotide metabolite sets, resulting in remarkable changes in the metabolic pathways of oxidative pentose phosphate pathway (oxPPP) and purine metabolism (Figure 3B). Functional enrichment analysis of the genes identified in both omics demonstrated that commonly downregulated genes in circNOLC1‐depleted cells were involved in the oxPPP (Figure S3D, Supporting Information). Further metabolomics analysis showed that the levels of intermediate metabolites in oxPPP and purine metabolism were significantly decreased after silencing circNOLC1 (Figure 3C). Some intermediate metabolites involved in PPP were confirmed by mass spectrometry, 6‐Phosphogluconic acid (6PG), Erythrose 4‐phosphate (E4P), and Gluconic acid were downregulated in circNOLC1‐depleted cells (Figure S3E, Supporting Information). Consistent with the metabolite levels, our qPCR findings demonstrated a significant decrease in the expression of key genes involved in the regulation of the oxPPP after silencing circNOLC1 (Figure 3D). Additionally, circNOLC1 knockdown led to a strong suppression of glucose consumption (Figure 3E). Previous studies indicated that PPP plays a critical role in regulating tumor growth and metastasis by supplying cells with not only ribose‐5‐phosphate but also nicotinamide adenine dinucleotide phosphate (NADPH) for detoxification of intracellular reactive oxygen species (ROS), reductive biosynthesis, and ribose biogenesis.^[^ ^15^, ^35^, ^36^, ^37^ ^]^ We found that circNOLC1 knockdown in HCT116 and LS174T cells robustly lessened NADPH level, raised the NADP^+^/NADPH ratio and the amount of intracellular ROS (Figure 3F,G). Furthermore, circNOLC1 silencing suppressed de novo DNA synthesis (Figure 3H). These results reveal that circNOLC1 is critical for regulating oxPPP.

G6PD is the rate‐limiting enzyme in the oxPPP, and its activity determines the first committed step of this pathway.^[^ ^37^ ^]^ Interestingly, circNOLC1‐depleted cells showed a strong reduction (∼55‐65%) in overall G6PD activity compared with control (Figure 3I). By integrated analysis of the RNA‐sequencing profile and the key genes involved in the regulation of the oxPPP, we found that the sterol regulatory element‐binding transcription factor 1 (SREBF1) level was significantly decreased in circNOLC1‐depleted cells (Figure 3J). SREBP, SREBF1‐encoded protein, is a transcription factor that activates genes encoding enzymes required for lipid synthesis. Thus, we examined the effect of circNOLC1 on lipid accumulation and found attenuated lipid levels in circNOLC1‐depleted cells compared with control (Figure 3K). In addition, SREBF1 deficiency led to strong reductions in the G6PD mRNA and protein levels (Figure S3F, Supporting Information). Because previous research indicated that mTOR complex 1 causes significant upregulation of G6PD by elevating the activity of SREBP1,^[^ ^16^, ^38^ ^]^ we determined whether circNOLC1 induced the expression of G6PD by controlling the levels of mTOR/SREBP. By qPCR and western blot analyses, we found that circNOLC1 knockdown dramatically suppressed mTOR, SREBP1, and G6PD expression (Figure 3L; Figure S3G, Supporting Information). Likewise, these mRNA and protein levels were significantly decreased in tumors formed in the livers of mice inoculated with cells expressing circNOLC1 shRNA compared with control (Figure 3M; Figure S3H, Supporting Information). Together, these results suggest that circNOLC1 enhances G6PD activity and, consequently, the oxPPP reprogramming and liver metastatic potential of CRC cells.

### 2.4 circNOLC1 Affects Glucose Uptake and mTOR/SREBP/G6PD Signaling by Interacting with AZGP1

**Figure 4:**
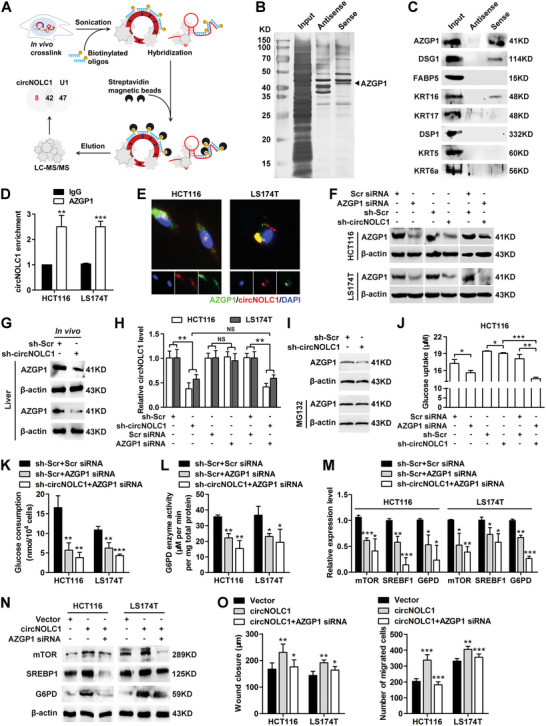
circNOLC1 affects glucose uptake and mTOR/SREBP/G6PD signaling by interacting with AZGP1. A) Schematic of the ChIRP‐MS workflow. B) Silver staining of proteins bound to the circNOLC1 sense (right lane) or antisense (middle lane) probes. The RNA pull‐down assay was performed with HCT116 cell lysates. A specific band (arrow) was identified as AZGP1 by mass spectrometry. C) AZGP1, DSG1, and KRT16 bound to circNOLC1 as confirmed by western blot after RNA pull‐down. D) CircNOLC1 bound to AZGP1 as shown by RIP followed by qPCR. E) Colocalization of circNOLC1 and AZGP1 was visualized by RNA FISH and IF staining in HCT116 and LS174T cells. Cy3 dye, FITC dye, and DAPI stain. F) Stable CRC cells expressing control or circNOLC1 shRNA were transfected with scramble or AZGP1 siRNA. Then cell lysates were applied to western blot analysis. G) Stable HCT116 cells expressing control or circNOLC1 shRNA were injected into the spleen. Liver tissue lysates were applied to western blot analysis. H) CRC cells were transfected as indicated, and the isolated total RNA was applied to qPCR. I) Effects of proteasome inhibitor MG132 on circNOLC1‐mediated AZGP1 protein level. HCT116 cells expressing control or circNOLC1 shRNA were treated with MG132 (20 µm) for 24 h. Then cell lysates were applied to western blot analysis. J) Glucose uptake level determined by Amplex Red assay in culture media from circNOLC1‐and/or AZGP1‐silenced HCT116 cells. Total amount of glucose in the media was used for normalization. K) Glucose consumption level in circNOLC1 and/or AZGP1 silenced CRC cells. Total cell number was used for normalization. L) G6PD activity in circNOLC1 and/or AZGP1 silenced CRC cells. M) The mRNA levels of mTOR, SREBP1, and G6PD in circNOLC1 and/or AZGP1 silenced CRC cells were determined by qPCR. N) The protein levels of mTOR, SREBP1, and G6PD were determined by western blot in circNOLC1‐overexpressing CRC cells after transfection with AZGP1 siRNA. O) The effect of circNOLC1 overexpression and/or AZGP1 knockdown on the migration ability of CRC cells was evaluated by wound healing (left) and Transwell (right) assays. ^*^ *p* < 0.05, ^**^ *p* < 0.01, ^***^ *p* < 0.001, NS, not significant. ChIRP, chromatin isolation by RNA purification; MS, mass spectrometry; RIP, RNA immunoprecipitation; FISH, fluorescence in situ hybridization; IF, immunofluorescence; siRNA, small interfering RNA; CRC, colorectal cancer; qPCR, quantitative reverse transcription PCR.

We next explored the molecular mechanism underlying circNOLC1‐induced oxPPP metabolism. Previous reports indicated that circRNAs can interact with proteins to form specific circRNPs that subsequently influence the function of associated proteins.^[^ ^39^ ^]^ Thus, we attempted to identify potential circNOLC1‐interacting proteins. We designed 4 probes against circNOLC1 and selectively retrieved over 95% of circNOLC1 from the cell by chromatin isolation by RNA purification (ChIRP). However, housekeeping genes GAPDH and β‐actin mRNA were no enriched (Figure S4A, Supporting Information). We then performed ChIRP assays in vitro with biotinylated circNOLC1 or U1, followed by mass spectrometry (**Figure** **4**A; Table S4, Supporting Information). U1 snRNAs are ideal for positive control to validate ChIRP‐MS because they are abundant and the spliceosome composition is well known.^[^ ^39^ ^]^ In total, we identified eight proteins that specifically interacted with circNOLC1 (Table S5, Supporting Information). Among them, three (AZGP1, DSG1, and KRT16) were confirmed to bind circNOLC1 by RNA pull‐down (Figure 4B,C; Figure S4B,C, Supporting Information). Since AZGP1 is highly expressed in CRC and plays an important role in glucose metabolism,^[^ ^40^, ^41^, ^42^ ^]^ we focused on this protein. By RNA immunoprecipitation (RIP) assay, we found that circNOLC1 was markedly enriched in pull‐downs with antibodies against AZGP1 (Figure 4D). Moreover, circNOLC1 colocalized with AZGP1 in the cytoplasm (Figure 4E). In addition, we predicted the interacting regions between circNOLC1 and AZGP1 by catRAPID algorithm (Figure S4D, Supporting Information) and generated a series of circNOLC1 deletion mutants to determine the nucleotide sequence of circNOLC1 that binds AZGP1 (Figure S4E, Supporting Information). Mutants containing nt 91 to 150 of circNOLC1 exhibited binding with AZGP1 relative to other mutants (Figure S4F, Supporting Information). Overexpression of nt 91–150 circNOLC1 substitution mutation remarkably reduced CRC cell proliferation and migration abilities compared with full‐length circNOLC1 (Figure S4G–J, Supporting Information), suggesting that nt 91 to 150 of circNOLC1 are required for the interaction of circNOLC1 with AZGP1.

Next, we used siRNA specifically targeting AZGP1 to reduce its expression level in HCT116 and LS174T cells (Figure S4K, Supporting Information). We observed a prominent reduction in AZGP1 protein expression upon circNOLC1 or AZGP1 knockdown and an increase in the expression of this protein when circNOLC1 was overexpressed (Figure 4F; Figure S4L, Supporting Information), without a corresponding change in AZGP1 mRNA level (Figure S4M, Supporting Information). AZGP1 protein levels were significantly decreased in tumors formed in the livers of mice inoculated with cells expressing circNOLC1 shRNA compared with control (Figure 4G). However, no significant difference in the circNOLC1 level was observed in AZGP1‐depleted cells (Figure 4H). We next treated HCT116 cells expressing control or circNOLC1 shRNA with a proteasome inhibitor and found that MG132 dramatically increased AZGP1 protein level (Figure 4I), suggesting that circNOLC1 hinders AZGP1 protein degradation.

Previous studies have shown that dysregulation of glucose homeostasis dramatically impacts PPP reprogramming and tumor metastasis.^[^ ^43^ ^]^ By measuring the amount of glucose, we found that knockdown of either circNOLC1 or AZGP1 alone inhibited glucose uptake and consumption. A further marked glucose reduction was observed after silencing both AZGP1 and circNOLC1 (Figure 4J,K; Figure S4N, Supporting Information). Moreover, AZGP1 silencing also enhanced the inhibitory effects of circNOLC1 knockdown on G6PD activity and expression (Figure 4L,M), further suggesting that regulation of the oxPPP by circNOLC1 is partially dependent on AZGP1. We next determined whether the effect of circNOLC1 on mTOR/SREBP signaling is associated with AZGP1. The combination of circNOLC1 and AZGP1 knockdown led to lower levels of mTOR, SREBP1, and G6PD than that observed with circNOLC1 or AZGP1 suppression alone in HCT116 and LS174T cells (Figure 4M; Figure S4O, Supporting Information). Furthermore, AZGP1 knockdown inhibited circNOLC1‐induced increases in mTOR, SREBP1, and G6PD protein levels (Figure 4N). Functional studies also demonstrated that AZGP1 downregulation abrogated the changes in the migratory potential of CRC cells induced by circNOLC1 overexpression (Figure 4O). Together, these results suggest that circNOLC1 can activate mTOR/SREBP/G6PD signaling through AZGP1 to enhance the PPP.

### 2.5 circNOLC1 Canonically Functions as a Sponge for miR‐212‐5p to Promote CRC Liver Metastasis by Upregulating c‐Met

**Figure 5:**
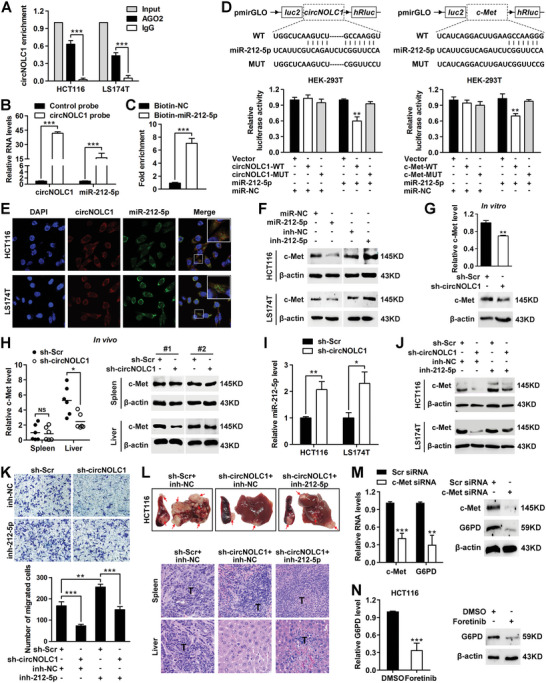
circNOLC1 canonically functions as a sponge for miR‐212‐5p to promote CRC liver metastasis by upregulating c‐Met. A) RIP assay using an antibody against AGO2 in extracts from HCT116 and LS174T cells. B) miR‐212‐5p was pulled down and enriched with a circNOLC1‐specific probe and was then detected by qPCR. C) The level of circNOLC1 in the streptavidin‐captured fractions from HCT116 cell lysates after transfection with biotinylated miR‐212‐5p or control RNA followed by qPCR. D) Luciferase activity assay for targeting sequences of the circNOLC1 or c‐Met 3′‐UTR by miR‐212‐5p in HEK‐293T cells. E) Colocalization of circNOLC1 and miR‐212‐5p was visualized by RNA FISH in HCT116 and LS174T cells. Cy3 dye, FAM dye, and DAPI stain. F) The protein levels of c‐Met were determined by western blot in CRC cells after transfection with miR‐212‐5p mimics or inhibitors. G) The mRNA and protein levels of c‐Met in circNOLC1‐silenced HCT116 cells were determined using qPCR and western blot analysis. H) Stable HCT116 cells expressing control or circNOLC1 shRNA were injected into the spleen. The isolated total RNAs and lysates of spleen and liver tissues were applied to qPCR (left) and western blot analysis (right). I) The expression levels of miR‐212‐5p in circNOLC1‐silenced CRC cells were determined using qPCR. J) c‐Met expression was measured by western blot analysis in circNOLC1‐silenced CRC cells after transfection with miR‐212‐5p inhibitors. K) The effect of miR‐212‐5p knockdown on the migration ability of circNOLC1‐silenced CRC cells was evaluated by Transwell assay. Quantitation of the indicated cells counted individually per visual field. L) The spleen and liver were examined at the gross anatomical level for tumors and metastases after intra‐splenic injection with circNOLC1 knockdown or overexpressed HCT116 cells. The red arrows indicate tumor foci. Representative H&E staining showing primary (upper) and metastatic (lower) nodules. T, tumor. M) HCT116 cells were transiently transfected scramble or c‐Met siRNA, and the isolated total RNAs and lysates were applied to qPCR and western blot analysis. N) G6PD levels in HCT116 cells were detected by qPCR and western blot after foretinib treatment for 24 h. ^*^ *p* < 0.05, ^**^ *p* < 0.01, ^***^ *p* < 0.001, NS, not significant. RIP, RNA immunoprecipitation; UTR, untranslated region; FISH, fluorescence in situ hybridization; siRNA, small interfering RNA; CRC, colorectal cancer; qPCR, quantitative reverse transcription PCR.

Given that circRNAs canonically function as miRNA sponges and that circNOLC1 is stable and localized in the cytoplasm, we investigated whether circNOLC1 can bind to miRNAs during CRC progression. We analyzed Argonaute 2 (AGO2) CLIP data (GSE28865) and found that circNOLC1 could bind to AGO2. AGO2 immunoprecipitation analysis further supported this observation (**Figure** **5**A). By bioinformatics analysis, we screened the miRNA targets of circNOLC1 and found that 7 potential miRNAs could bind to circNOLC1 (Figure S5A, Supporting Information). Among these miRNAs, miR‐212‐5p was the only tumor suppressor previously reported to be implicated in CRC metastasis (Table S6, Supporting Information), and its expression levels were significantly downregulated in CRC cell lines (Figure S5B, Supporting Information). By circRNA in vivo immunoprecipitation (circRIP) assay, we found specific enrichment of circNOLC1 and miR‐212‐5p in the complex compared with control (Figure 5B; Figure S5C, Supporting Information). Moreover, by miRNA pull‐down assay with biotin‐coupled miR‐212‐5p mimics, circNOLC1 was efficiently enriched by miR‐212‐5p (Figure 5C). In addition, we found that both circNOLC1 and c‐Met 3′‐UTRs share common miRNA response elements (MREs) of miR‐212‐5p, which might suggest an association among circNOLC1, miR‐212‐5p, and c‐Met in CRC. Thus, we performed luciferase reporter assays and revealed that cotransfection of miR‐212‐5p markedly reduced the luciferase activity of circNOLC1 or c‐Met 3′‐UTR wild‐type reporter by at least 40%, whereas had no effect on the mutant one (Figure 5D). RNA‐FISH results further confirmed that circNOLC1 colocalized and interacted with miR‐212‐5p (Figure 5E). Moreover, the c‐Met level was decreased in CRC cells after transfection with miR‐212‐5p mimics but increased following treatment with miR‐212‐5p inhibitors (Figure 5F; Figure S5D, Supporting Information).

To further validate the promotive effect of circNOLC1 on c‐Met, we detected the expression of c‐Met after silencing circNOLC1 and found that the c‐Met mRNA and protein levels were strikingly downregulated (Figure 5G). A similar c‐Met decrease was observed in the livers of mice inoculated with cells expressing circNOLC1 shRNA, but no differences were observed in the spleens of these mice (Figure 5H; Figure S5E, Supporting Information). In addition, miR‐212‐5p expression significantly increased after silencing circNOLC1 (Figure 5I), whereas circNOLC1 showed no significant changes after miR‐212‐5p overexpression (Figure S5F, Supporting Information). Therefore, we presumed that circNOLC1 modulates CRC liver metastasis mainly by protecting c‐Met from downregulation by miR‐212‐5p. As expected, circNOLC1 knockdown suppressed c‐Met expression, this effect was reversed after silencing miR‐212‐5p (Figure 5J). In addition, we observed a prominent reduction in c‐Met expression upon nt 61–67 circNOLC1 substitution mutation overexpression (Figure S5G, Supporting Information). Functionally, miR‐212‐5p inhibitor could restore circNOLC1 silencing‐suppressed migratory abilities of CRC cells (Figure 5K). In vivo analysis demonstrated that the number of metastatic nodules in the liver decreased after being inoculated with the circNOLC1‐depleted HCT116 cells and were largely restored by miR‐212‐5p inhibitor (Figure 5L).

Previous research indicated that c‐Met inhibition can elicit metabolic reprogramming, involving disorders of glycolysis and PPP.^[^ ^44^ ^]^ By using foretinib or siRNA‐specific knockdown of c‐Met, we found that inhibition of c‐Met signaling significantly decreased G6PD expression level in HCT116 cells (Figure 5M,N). We next used the CRISPRi dCas9‐KRAB system to silence G6PD expression. However, no significant difference in c‐Met mRNA and protein levels was observed between HCT116 cells expressing control and dCas9‐KRAB‐sgRNA‐G6PD (Figure S5H, Supporting Information), implicating that G6PD is a functional downstream gene of c‐Met in CRC cells. Together, these results suggest that circNOLC1 canonically functions as a sponge for miR‐212‐5p to upregulate c‐Met, this signaling was indeed responsible for G6PD‐induced oxPPP in CRC liver metastasis.

### 2.6 YY1 Regulates circNOLC1 Expression, and Upregulated circNOLC1 Stabilizes Its Parental Gene NOLC1 mRNA Through HuR

**Figure 6:**
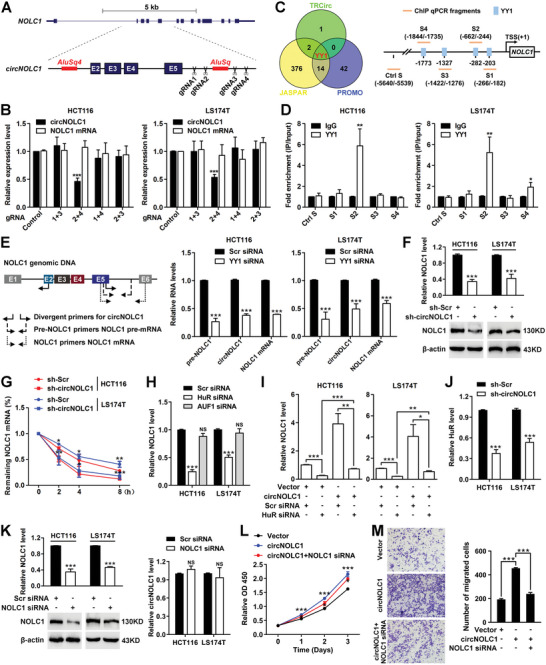
YY1 regulates circNOLC1 expression, and upregulated circNOLC1 stabilizes its parental gene NOLC1 mRNA through HuR. A) Schematics showing that the genomic region of NOLC1 exons 2–5 contains highly complementary flanking Alu repeats and long introns. AluSq elements and long introns were deleted using the CRISPR/Cas9 system. Primers flanking gRNA recognition sites were used to detect deletions. B) CRISPR/Cas9‐mediated genomic deletions in CRC cells as determined by qPCR. Four pairs of gRNAs (gRNA 1+3, gRNA 2+4, gRNA 1+4, gRNA 2+3) were used to mediate deletion of the AluSq element. C) Three independent TF target databases were used to predict the potential TFs binding to the promoter of NOLC1 (left). Schematic of the human NOLC1 gene and its promoter region (right). D) qPCR of the ChIP products validated the binding capacity of YY1 to the NOLC1 promoter. E) Schematic of the primers used for distinguishing between circNOLC1, pre‐NOLC1, and NOLC1 mRNA (left). HCT116 and LS174T cells were transiently transfected with scramble or YY1 siRNA, and genomic DNA was isolated and applied to qPCR (right). F) The mRNA and protein levels of NOLC1 in circNOLC1‐silenced CRC cells were determined using qPCR and western blot analysis. G) NOLC1 level in circNOLC1‐silenced CRC cells was determined by qPCR after actinomycin D treatment for 2, 4, and 8 h. H) HCT116 and LS174T cells were transiently transfected with HuR or AUF1 siRNA, and the isolated total RNA was applied to qPCR. I) The mRNA level of NOLC1 in circNOLC1‐overexpressing and/or HuR‐silenced CRC cells was determined by qPCR. J) HuR expression was detected by qPCR in circNOLC1‐silenced HCT116 and LS174T cells. K) Efficient knockdown of NOLC1 in HCT116 and LS174T cells was detected by qPCR and western blot (left). The circNOLC1 level in CRC cells was determined by qPCR after transfection with scramble or NOLC1 siRNA (right). L) The effect of circNOLC1 overexpression and/or NOLC1 knockdown on the proliferation ability of CRC cells was evaluated by CCK8 assays. M) The effect of circNOLC1 overexpression and/or NOLC1 knockdown on the migration ability of CRC cells was evaluated by Transwell assays. ^*^ *p* < 0.05, ^**^ *p* < 0.01, ^***^ *p* < 0.001, NS, not significant. CRISPR, clustered regularly interspaced short palindromic repeats; gRNA, guide RNA; TF, transcription factor; TSS, transcriptional start site; siRNA, small interfering RNA; CRC, colorectal cancer; qPCR, quantitative reverse transcription PCR.

We next evaluated why circNOLC1 was formed and upregulated in CRC. It was previously reported that the inverted Alu repeats on long flanking introns can pair to form RNA duplexes, which significantly enhances back‐splicing, and thus contribute to circRNA biogenesis.^[^ ^45^ ^]^ Therefore, we evaluated whether circNOLC1 formation was promoted by this mechanism. Bioinformatics analysis showed that the flanking introns of NOLC1 (exons 2–5) contained highly complementary Alu repeats with fourteen short interspersed elements (SINEs) in intron 1 and one SINE (named AluSq) in intron 5 (Figure S6A, Supporting Information). By using NCBI BLAST, we aligned the sequence of intron 1 to that of intron 5 of the NOLC1 gene and found the AluSq4 (in intron 1) and AluSq (in intron 5) elements were highly reverse complementarity (67% identity over 299 nucleotides, Figure S6B, Supporting Information), implying that the two inverted complementary sequences may mediate the circularization process of circNOLC1. To test this possibility, we designed four pairs of guides RNAs directed at the boundary of the AluSq sequence (**Figure** **6**A; Figure S6C, Supporting Information), each gRNA pair was cotransfected with the Cas9 expression vector. By qPCR analysis, we determined the efficiencies of the guide RNAs and found that the expression level of circNOLC1 was significantly decreased in gRNA2+4 (Figure 6B). However, this deletion did not influence the expression of NOLC1 mRNA, indicating that the long flanking introns AluSq and AluSq4 are indispensable for circNOLC1 formation.

As both circRNAs and mRNAs are derived from precursor mRNAs (pre‐mRNAs),^[^ ^45^, ^46^, ^47^ ^]^ and circRNA biogenesis can be regulated by transcription factors (TFs),^[^ ^48^ ^]^ we assumed that upregulation of circNOLC1 in CRC may be transcriptionally regulated by certain TFs. We used three independent databases to predict TFs that may be involved and found that the promoter of NOLC1 contains some binding motifs of YY1 (Figure 6C; Table S7, Supporting Information). ChIP analysis validated the direct binding of YY1 to the NOLC1 promoter (Figure 6D). By analyzing TPM in the TCGA database, we found that both YY1 and NOLC1 were upregulated in CRC compared with normal samples, and levels of YY1 and NOLC1 exhibited a strong positive correlation (Figure S6D, Supporting Information). Moreover, YY1 knockdown with siRNA in HCT116 and LS174T cells (Figure S6E,F, Supporting Information) significantly reduced levels of both circNOLC1 and mature NOLC1 mRNA while inducing variable reductions in the pre‐NOLC1 RNA level (Figure 6E), suggesting that the upregulation of circNOLC1 in CRC cells results at least partially from elevated YY1 expression.

Cells were transduced with lentiviral vectors containing short hairpin RNA complementary to the backsplicing junction of circNOLC1. Endogenous circNOLC1, but not NOLC1, could be successfully depleted since the backsplicing junction is the only unique and targetable feature. Interestingly, circNOLC1 knockdown led to substantially reduced mRNA and protein levels of NOLC1 (Figure 6F). We speculated that the downregulation of NOLC1 expression is not attributed to the transfection of shRNAs targeting circNOLC1 but may be subject to other post‐transcriptional regulation. By assessing NOLC1 mRNA stability, we found that silencing circNOLC1 shortened the half‐life of NOLC1 mRNA by ≈2 h (Figure 6G). RNA binding proteins, such as HuR and AUF1, have been reported to broadly regulate RNA stabilization in CRC. We found that after silencing HuR but not AUF1, NOLC1 expression was obviously downregulated in both HCT116 and LS174T cells (Figure 6H; Figure S6G, Supporting Information). Silencing HuR could significantly reverse the NOLC1 mRNA level induced by circNOLC1 overexpression (Figure 6I). Furthermore, HuR expression was diminished when circNOLC1 was depleted (Figure 6J). NOLC1 knockdown did not change the circNOLC1 level (Figure 6K). Silencing NOLC1 can markedly change circNOLC1 overexpression‐induced the proliferation and migration abilities of CRC cells (Figure 6L,M). Together, these results suggest that circNOLC1 partially enhances the stability of its parental gene NOLC1 mRNA by promoting HuR expression.

### 2.7 circNOLC1 Expression is Positively Correlated with G6PD and c‐Met Expression but Negatively Correlated with miR‐212‐5p Expression in Human CRC Specimens

**Figure 7:**
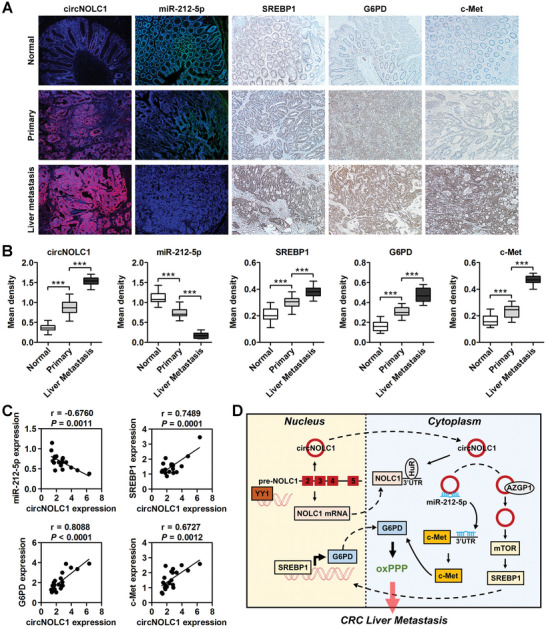
circNOLC1 expression is positively correlated with G6PD and c‐Met expression but negatively correlated with miR‐212‐5p expression in human CRC specimens. A) Representative FISH images of circNOLC1 and miR‐212‐5p, and the corresponding IHC images of SREBP1, G6PD, and c‐Met staining in representative samples of normal colorectal mucosa, primary CRC, and matched liver metastases from 20 CRC patients. B) Semiquantitative analysis of the expression levels of circNOLC1, miR‐212‐5p, SREBP1, G6PD, and c‐Met in twenty pairs of matched human normal colorectal mucosa, primary CRC, and corresponding liver metastasis samples. C) Correlation between circNOLC1 level and the expression of miR‐212‐5p, SREBP1, G6PD, and c‐Met in CRC samples. The Pearson correlation coefficient (r) with the respective significance is indicated. D) Schematic representation of the putative roles of the circNOLC1‐oriented regulatory network in CRC liver metastases. ^***^ *p* < 0.001. FISH, fluorescence in situ hybridization; IHC, immunohistochemistry; CRC, colorectal cancer.

To determine the clinical relevance of our observations, we determined whether there are correlations between circNOLC1, miR‐212‐5p, SREBP1, G6PD, and c‐Met expression in primary CRC and liver metastasis (Table S8, Supporting Information). By FISH and IHC staining, we found that the circNOLC1 signal was increased and the miR‐212‐5p signal was reduced in liver metastasis compared with their corresponding primary CRC. In these same samples, there was increased SREBP1, G6PD, and c‐Met staining (**Figure** **7**A,B), consistent with the differential analysis of the TCGA‐COADREAD cohorts (Figure S7A, Supporting Information). Analysis of the unmatched 63 normal, 194 primary CRC, and 53 liver metastatic samples from GEO: GSE54088 and GSE68468, respectively, confirmed that SREBP1, G6PD, and c‐Met were upregulated, while miR‐212‐5p was downregulated in liver metastasis samples (Figure S7B, Supporting Information). Analysis of the liver metastasis and normal liver samples in these GEO datasets suggested that G6PD and c‐Met levels were even higher in liver metastasis than in normal liver samples (Figure S7C, Supporting Information). In addition, a direct correlation between SREBP1, G6PD, and c‐Met levels and the circNOLC1 level, as well as an inverse correlation between the levels of miR‐212‐5p and circNOLC1, were detected (Figure 7C). These clinical data support our preclinical findings and suggest that upregulation of circNOLC1 occurs in the setting of liver metastasis in CRC patients, and this upregulation may coincide with dysregulation of glucose homeostasis associated with the PPP reprogramming.

## 3 Discussion

Metabolic reprogramming contributes to tumor development and metastasis. Activation or overexpression of tumor‐promoting genes, continuous activation of related signaling pathways, and inactivation of tumor suppressor genes are the main mechanisms that lead to tumor cell metabolic reprogramming.^[^ ^49^ ^]^ Here, we described circNOLC1 and evaluated its functional role in the regulation of metabolic reprogramming in colon cancer.

circNOLC1 was first reported to have important regulatory potency with tissue/developmental‐stage‐specific expression^[^ ^50^ ^]^ and later found to be highly expressed in the human colon.^[^ ^51^ ^]^ In this study, we found that circNOLC1 was highly abundant in both human CRC cell lines and clinical samples. The striking upregulation of circNOLC1 particularly occurred in the setting of liver metastasis. Analysis of the TCGA‐COADREAD cohorts also revealed the stage‐dependent increase of circNOLC1 expression. In addition, circNOLC1 silencing specifically impairs the growth and liver metastasis of CRC cells in vitro and in vivo. These data support the hypothesis that circNOLC1 may play a critical role in CRC liver metastasis. It is consistent with previous findings that upregulation of circNOLC1 expression is associated with a malignant progression in other types of tumors.^[^ ^52^, ^53^, ^54^ ^]^

CircRNAs, a unique class of noncoding RNAs, are emerging as key regulators of metabolic reprogramming. It has been reported that the cellular level of circACC1 modulates both fatty acid β‐oxidation and glycolysis, resulting in profound changes in cellular lipid storage.^[^ ^21^ ^]^ The circ‐CUX1/EWSR1/MAZ axis is a therapeutic target for aerobic glycolysis and neuroblastoma progression.^[^ ^55^ ^]^ In this study, we found that circNOLC1 regulates metabolic reprogramming in colon cancer cells by promoting oxPPP reprogramming. The mTOR pathway, a key signaling pathway for initiating metabolic reprogramming,^[^ ^16^ ^]^ is closely related to the occurrence and development of colon cancer.^[^ ^56^ ^]^ It also promotes the expression of G6PD in an SREBF1‐dependent manner in HEK293 cells. Our results show that the ability of circNOLC1 to affect the activities of SREBP1 and G6PD, which in turn affects the PPP, is dependent on mTOR. Nevertheless, we cannot exclude the possibility that circNOLC1 contributes to CRC liver metastasis through metabolic pathways other than PPP. For example, mTOR can regulate glycolysis and lipid synthesis in the cancer progression.^[^ ^38^, ^57^ ^]^ We found that the knockdown of circNOLC1 decreased levels of SREBF1 mRNA and SREBP protein, a transcription factor that regulates lipid synthesis, and attenuated lipid accumulation. However, we observed no difference in glycolytic and lipid metabolites between control and circNOLC1‐depleted CRC cells. We also have not excluded an effect of circNOLC1 on pathways other than mTOR signaling.

CircRNAs can interact with different proteins to form specific circRNPs that subsequently influence the modes of action of associated proteins.^[^ ^58^ ^]^ CircCTIC1 interacts with the nuclear remodeling factor complex, recruits the complex to the c‐Myc promoter, and finally drives the initiation of c‐Myc transcription.^[^ ^59^ ^]^ The interaction of circPTK2 and vimentin mediates the regulation of CRC, as demonstrated by knockdown or overexpression of vimentin.^[^ ^60^ ^]^ In this study, CHIRP‐MS was used to analyze the possible binding proteins of circNOLC1 and RIP and RNA pulldown assays were used to further identify AZGP1 as its interacting protein. A previous study reported that AZGP1 regulates CRC cell metastasis by interacting with FLNA and regulating the focal adhesion pathway.^[^ ^40^ ^]^ Our results provide biochemical evidence that circNOLC1 interacts with AZGP1 and facilitates CRC cell proliferation and migration. In addition, the exact binding site was explored. We found that nt 91–150 regions of circNOLC1 can recruit AZGP1 and consequently affect glucose uptake and mTOR/SREBP/G6PD signaling. It is consistent with previous findings that AZGP1 directly stimulates glucose uptake into skeletal muscle in vitro.^[^ ^41^ ^]^ Our results indicate that circNOLC1 interacts with AZGP1 to promote liver metastasis of colon cancer by reprogramming the oxPPP.

circRNAs can regulate their host genes at the transcriptional and post‐transcriptional levels. It is reported that circFLI1 binds to the FLI1 promoter and induces DNA demethylation, which epigenetically activates FLI1, in breast cancer.^[^ ^61^ ^]^ Circ‐ENO1 acts as a ceRNA to interact with miR‐22‐3p and upregulate ENO1 expression in lung adenocarcinoma.^[^ ^62^ ^]^ In this study, we showed that circNOLC1 can upregulate the expression of NOLC1 by maintaining the stability of NOLC1 mRNA through RNA binding protein HuR. It is consistent with previous findings that circRNAs regulate gene expression by regulating or interacting with HuR. Circ‐CCND1 binds to the HuR protein to upregulate CCND1 mRNA expression.^[^ ^63^ ^]^ CircSHKBP1 sponges miR‐582‐3p to increase HuR expression, enhance VEGF mRNA stability, and induce angiogenesis.^[^ ^64^ ^]^

In summary, we identified a novel pathway underlying CRC liver metastasis by circNOLC1‐mediated oxPPP reprogramming. This signaling may contribute to an improved understanding of malignant progress in CRC. These results may also have implications for the clinical treatment of patients with CRC liver metastasis.

## 4 Experimental Section

### Patients and Samples

Colorectal carcinoma tissues, corresponding adjacent normal tissues, and matched liver metastatic tissues were obtained from surgical resections of patients without preoperative treatment at the Second Hospital of Dalian Medical University (Dalian, China) between 2015 and 2019. With approval and support from the ethics committee of the Second Hospital of Dalian Medical University, the diagnosis of colorectal carcinoma was confirmed by pathologic examination. Informed consent was obtained from all patients. The samples used in the circRNA microarray and qPCR analyses were freshly frozen, and the samples used for FISH and IHC staining were FFPE (formalin‐fixed and paraffin‐embedded).

### Total RNA Extraction, RNase R Treatment, and circRNA Microarray

Total RNA was extracted from three pairs of colon carcinoma and adjacent normal tissues using TRIzol (Invitrogen) and treated with RNase R (Epicentre). Microarray analysis was performed by KangChen Biotech (Shanghai, China). Briefly, the enriched circular RNAs were amplified and transcribed into fluorescent cRNAs utilizing Arraystar Super RNA Labeling Kit (Arraystar). The labeled cRNAs were purified by RNeasy Mini Kit (Qiagen) and hybridized to Arraystar Human circRNA Array V2 (8×15K, Arraystar). Subsequently, the arrays were scanned using an Agilent G2505C Microarray Scanner, and acquired array images were analyzed with Agilent Feature Extraction software.

### In Vivo Metastasis

The animal studies were approved by the Animal Center of Dalian Medical University in accordance with the national guidelines for the care and use of laboratory animals. BALB/c athymic nude mice that were 4–6 weeks of age were purchased from Charles River Laboratories (Beijing, China). The liver metastasis model was implemented as the previously described method.^[^ ^30^, ^31^ ^]^ Briefly, 2 × 10^6^ CRC cells (HCT116, LoVo, or LS174T) transduced with lentiviral vectors were injected into the spleens or liver capsule (Glisson's capsule) of recipient mice. The spleen or liver was returned to the abdominal cavity and the incision was closed. Mice were sacrificed at 3–7 weeks post‐injection and examined microscopically by H&E staining for the development of liver metastatic foci. The dissected tumors were collected for RNA and protein isolation.

### Mass Spectrometry Metabolomics Analysis

Stable HCT116 cells expressing control shRNA or circNOLC1 shRNA were seeded in 6 cm plates, and 2 h before metabolite collection, the culture medium was replaced with fresh medium. Widely targeted metabolomics analysis was performed by MetWare Biotechnology (Wuhan, China). Cells were thawed on ice and dissolved in a cold 70% methanol aqueous solution. Each sample was frozen for 3 min in liquid nitrogen and centrifuged for 2 min, repeating two times. The mixture was centrifuged under 12 000 r min^−1^ at 4 °C for 10 min. The supernatant was injected into chromatography columns for LC–MS/MS analysis. The extracts were analyzed using a UPLC–ESI–MS/MS system (Shim‐pack UFLC SHIMADZU CBM30A system; MS, QTRAP 6500+ System). Quantification of metabolites was accomplished using multiple reaction monitoring (MRM) analysis of a triple quadrupole‐linear ion trap mass spectrometer (QTRAP 6500+ System). For statistical analysis, the relative abundances of each metabolite were log‐transformed before analysis to meet normality. Differentially expressed metabolites were identified using a combination of the fold change values and the VIP values from the OPLS‐DA model. Metabolites with VIP values ≥1 and fold change ≥ 1.5 or ≤ 0.67 were considered differentially expressed.

### Chromatin Isolation by RNA Purification (ChIRP)‐Mass Spectrometry (MS)

For affinity capture of complexes containing circNOLC1 and chromatin, four probes covering the sequence of circNOLC1 for use in ChIRP were designed. U1 snRNA probes were used as the positive control. ChIRP‐MS analysis was performed by KangChen Bio‐tech (Shanghai, China). Briefly, 10^7^ cells were applied to reversible formaldehyde crosslinking in situ, followed by hybridization with biotin‐labeled probes. After washing to remove the nonspecifically bound proteins under strong denaturing conditions, the proteins that specifically interacted with circNOLC1 or control were identified and quantitatively analyzed by MS. Proteins with unique peptides ≥ 2 and fold change ≥ 1 were considered differentially expressed.

### RNA Pull‐Down Assays

RNA pull‐down assays were performed using a Magnetic RNA‐Protein Pull‐Down Kit (Thermo Scientific). circNOLC1 antisense and sense probes were synthesized with BiotinTEG at the 3′ end and purchased from Sangon Biotech. The biotinylated probes were incubated with nucleic acid‐compatible streptavidin magnetic beads at 4 °C for 30 min. These complexes were mixed with proteins extracted from CRC cells at 4 °C for 1 h with gentle rotation. After that, the beads were washed three times and eluted with an SDS loading buffer. Proteins were applied to SDS‐PAGE followed by silver staining or western blot analysis.

### RNA Immunoprecipitation

RNA immunoprecipitation assays were performed using an EZ‐Magna RIP Assay Kit (Millipore) according to the manufacturer's protocol. Briefly, cells were lysed in complete RIP lysis buffer, and the extracts were incubated with magnetic beads conjugated to antibodies or nonspecific IgG at 4 °C overnight. The antibody‐protein‐RNA complexes were isolated by immunoprecipitation with protein A/G magnetic beads. After that, the complexes were washed and incubated with proteinase K to remove proteins. Finally, RNAs were eluted and purified for RNA extraction and detection.

### Integrated Analysis of RNA‐seq and Metabolomics

The significantly differentially expressed genes (*q* value < 0.05) were identified from transcriptomics and integrated with the significantly differentially metabolites (VIP values ≥ 1) from metabolomics data. The integrated gene‐metabolite profile was analyzed with the Joint Pathway Analysis module of MetaboAnalyst (http://www.metaboanalyst.ca). Pearson correlation coefficients of metabolites and genes were calculated using an R package.

### Statistical Analysis

All results were presented as mean ± SD. Statistical analysis was performed by the SPSS 17.0 (SPSS) and GraphPad Prism 7. Student's *t*‐test, ANOVA, Chi‐square test, and Pearson correlation coefficients were used according to the type of experiment. *p* values < 0.05 were considered statistically significant. All patient tissues and clinical information were collected using protocols approved by the Ethics Committee of the Second Hospital of Dalian Medical University. Written informed consent was obtained from each patient. All animal procedures used were approved by the Animal Ethics Committee of Dalian Medical University and conducted according to the national guidelines for the care and use of laboratory animals.

## Conflict of Interest

The authors declare no conflict of interest.

## Author Contributions

M.Y., X.Z., F.Y., and F.Z. contributed equally to this work. S.R. and Y.Z. were responsible for the study concept and design. M.Y., F.Y., F.Z., S.J., X.Z., J.L., Z.Z., Y.S., Z.C., H.W., X.L., X.Y., B.W., K.J., and F.L. were responsible for performing the experiments, acquisition and analysis of data, and drafting the manuscript. X.Z. provided and collected the clinical data. Y.Z. and S.R. were responsible for study supervision. S.R., Y.Z., and M.Y. were responsible for critical revision of the manuscript.

## Supporting information

Supporting Information

## Data Availability

The data that support the findings of this study are available in the supplementary material of this article.
