# Correction to “CircNOLC1 Promotes Colorectal Cancer Liver Metastasis by Interacting with AZGP1 and Sponging miR‐212‐5p to Regulate Reprogramming of the Oxidative Pentose Phosphate Pathway”

**DOI:** 10.1002/advs.202414965

**Published:** 2025-04-26

Yuan, M., Zhang, X., Yue, F., Zhang, F., Jiang, S., Zhou, X., Lv, J., Zhang, Z., Sun, Y., Chen, Z., et al. (2023). CircNOLC1 Promotes Colorectal Cancer Liver Metastasis by Interacting with AZGP1 and Sponging miR‐212‐5p to Regulate Reprogramming of the Oxidative Pentose Phosphate Pathway. *Adv. Sci. 10*, e2205229. https://doi.org/10.1002/advs.202205229

The authors noticed that there was an error in Figure 6M. An incorrect image was used in Vector image. We have carefully reexamined all figures in the main document and Supporting Information. The authors confirm all results and conclusions of this article remain unchanged. We apologize for this error.

**Figure 6:**
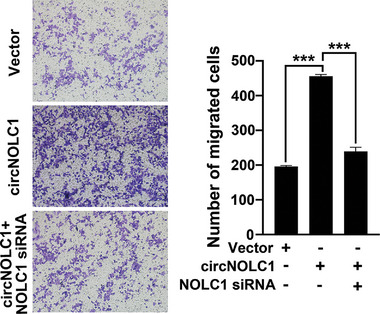
M) The effect of circNOLC1 overexpression and/or NOLC1 knockdown on the migration ability of CRC cells was evaluated by Transwell assays.

The correct image is shown above.

