# Supplementary material for: CircNOLC1 Promotes Colorectal Cancer Liver Metastasis by Interacting with AZGP1 and Sponging miR‐212‐5p to Regulate Reprogramming of the Oxidative Pentose Phosphate Pathway
Source: Adv Sci (Weinh). 2023 Oct 23;10(33):2205229. doi: 10.1002/advs.202205229 (PMC10667818; doi:10.1002/advs.202205229)
Supplement: Supplementary file 1 — Supporting Information [file ADVS-10-2205229-s001.pdf]

ADVANCED SCIENCE
Open Access

Supporting Information

for *Adv. Sci.*, DOI 10.1002/advs.202205229

CircNOLC1 Promotes Colorectal Cancer Liver Metastasis by Interacting with AZGP1 and Sponging miR-212-5p to Regulate Reprogramming of the Oxidative Pentose Phosphate Pathway

Menglang Yuan, Xinsheng Zhang, Fangxia Yue, Feifan Zhang, Sufen Jiang, Xu Zhou, Jinjuan Lv, Zhenyu Zhang, Yuzhu Sun, Zihao Chen, Han Wu, Xiaoqian Liu, Xiaoqi Yu, Bowen Wei, Kexin Jiang, Fang Lin, Yunfei Zuo and Shuangyi Ren**

Supporting Information

CircNOLC1 promotes colorectal cancer liver metastasis by interacting with AZGP1 and sponging miR-212-5p to regulate reprogramming of the oxidative pentose phosphate pathway

Menglang Yuan[1,3☆]; Xinsheng Zhang[1☆]; Fangxia Yue[2☆]; Feifan Zhang[1☆]; Sufen Jiang[2]; Xu Zhou[2]; Jinjuan Lv[2]; Zhenyu Zhang[2]; Yuzhu Sun[2]; Zihao Chen[1]; Han Wu[2]; Xiaoqian Liu[2]; Xiaoqi Yu[2]; Bowen Wei[2]; Kexin Jiang[2]; Fang Lin[2]; Yunfei Zuo[2*]; Shuangyi Ren[1*]

This file includes:

Supplementary Materials and Methods

Supplementary Tables

Supplementary Figures and Figure legends

Supplementary Materials and Methods

Cell culture

Human colon cancer (HCT116, LS174T, LoVo, and SW620) and HEK-293T cell lines were purchased from the Institute of Biochemistry and Cell Biology of the Chinese Academy of Sciences (Shanghai, China), and normal human intestinal epithelial cells (HIECs and NCM460) were obtained from Xijing Hospital of Digestive Diseases (Xian, China). Human normal hepatocyte (L-02) and lung epithelial cells (BEAS-2B) cell lines were purchased from iCell Bioscience Inc (Shanghai, China). Cells were cultured in RPMI-1640 medium or Dulbecco's modified Eagle's medium supplemented with 10% fetal bovine serum (Gibco) and 100 U/ml penicillin-streptomycin solution at 37°C in 5% CO_2.

Plasmid construction, siRNA treatment, and lentiviral transduction

The parallel DNA sequences for shRNA were synthesized and cloned into the pGLVU6-GFP vector. Lentiviruses for gene knockdown or overexpression experiments were purchased from GenePharma (Shanghai, China). For stable circNOLC1 knockdown or overexpression, cells were transduced with lentiviral vectors containing circNOLC1 short hairpin RNA (shRNA) or overexpression, and clones were selected with 2 mg/ml puromycin (Sigma–Aldrich). circNOLC1 wild-type, nt 91-150 (with AZGP1 binding site) or nt 61-67 (with miR-212-5p binding site) substitution mutant plasmids were synthesized from PPL (Nanjing, China). The circular RNA fragment was synthesized by two sets of primers with restriction sites (BamHI and EcoRI) and inserted into the pLenti-CMV-CircRNA-EF1-GFP-Puro vector. For the CRISPR/Cas9 assay, sgRNA cloning vectors were constructed using LentiCRISPRv2 cloning vector. dCas9-KRAB plasmid (ID: 92361) was obtained from Addgene. All constructs were verified by sequencing. SREBF1, AZGP1, YY1, HuR, AUF1, and NOLC1 small interfering RNAs (siRNAs), miR-212-5p mimics and inhibitors were synthesized by GenePharma. The transient transfection of siRNAs, miRNA mimics and plasmids were carried out using Lipofectamine 2000 (Invitrogen) following the manufacturer's instructions.

Reverse transcription and quantitative reverse transcription PCR

Total RNA was isolated from cultured cells and fresh tissues using TRIzol (Invitrogen). The concentrations of RNA were determined using a NanoDrop 2000 (Thermo Scientific). For validation of circular RNA expression, RNA was reverse transcribed with random hexamer primers (Takara) for detection of linear and circular RNA transcripts and with oligo$(dT)_{18}$ primers (Thermo Scientific) for detection of linear transcripts containing poly(A) tails. For circRNA and mRNA analyses, RNA was reverse transcribed into cDNA by using a PrimeScript RT Reagent Kit (Takara). qPCR primers were synthesized from Sangon Biotech (Shanghai, China), and the reaction was performed using ChamQ Universal SYBR qPCR Master Mix (Vazyme). circRNA and mRNA expression were normalized to GAPDH expression. For miRNA analyses, qPCR was performed using a miRcute Plus miRNA qPCR Kit (TIANGEN) and commercially available primers (TIANGEN). miRNA expression was normalized to U6 expression. All of the primers used are listed in Supplementary Table S10.

Actinomycin D assay

To assess RNA stability, cells were incubated with 2 or 5 μg/ml actinomycin D (MedChemExpress) for different time points. After that, cells were harvested and the relative RNA levels were analyzed by qPCR and normalized to the values measured in the 0 h group.

Cytoplasmic and nuclear RNA isolation

The subcellular localization of circNOLC1 was detected using a PARIS Kit (Life Technologies) according to the manufacturer's instructions.

Sanger sequencing

cDNA from HCT116 cells was amplified by PCR using primers specifically targeting circNOLC1. To confirm head-to-tail splicing, Sanger sequencing was conducted with the sequencing primer F:5'-TCCGAGGATGAGCCACCA-3';
R:5'-ACTTTCGCTCTGGGACCTT-3'.

Western blot

Total protein was extracted from cells and tissues using a Whole Cell Lysis Assay Kit (KeyGen). Equal amounts of protein were resolved by SDS–PAGE and transferred onto

nitrocellulose filter membranes (Life Science). The membranes were probed with primary antibodies, followed by washing and incubation with horseradish peroxidase-conjugated secondary antibodies. Antigen-antibody complexes were visualized with enhanced chemiluminescence (Advansta) and image analyzer ImageQuant LAS 500 (GE Healthcare).

RNA fluorescence in situ hybridization (FISH)

Cy3-labeled circNOLC1 and FAM-labeled hsa-miR-212-5p probes were purchased from GenePharma. The sequence of the probe for circNOLC1 was 5'-Cy3-CAUCC UUGCU GUGUC UGGUU UGGGA GGG-3', and the sequence of the probe for miR-212-5p was 5'-FAM-AGTAA GCAGT CTAGA GCCAA GGT-3'. FISH assays were performed using a Fluorescence In Situ Hybridization Kit (GenePharma) according to the manufacturer's instructions. Cells were processed and co-incubated with probes at 37°C for 16h. Nuclei were stained with 4',6'-diamidino-2-phenylindole (DAPI, Solarbio). Fluorescence signals were detected and visualized at RT using a Leica TCS SP5II scanning confocal microscope (Leica). FFPE sections were dewaxed in xylene and gradually rehydrated with gradient alcohol. After digestion with 40 μg/ml proteinase K, hybridization was carried out at 37°C overnight in a dark moisture chamber. The slides were washed three times in 50% formamide/2×SSC for 5 min respectively. Images were acquired using a fluorescence microscope (Olympus). The tissues were scored by quantifying the fluorescence density in 10 separate fields in the areas with the highest fluorescence intensity.

Immunofluorescence and confocal microscopy analyses

Cells were rinsed twice with cold PBS and fixed with 4% paraformaldehyde for 15 min at RT. Nonspecific binding was blocked with 5% goat serum for 30 min at RT. Then, cells were incubated with a rabbit anti-human AZGP1 antibody (Proteintech) diluted in PBS. After overnight incubation at 4°C, cells were incubated with FITC-conjugated secondary antibodies (Solarbio) for 1.5 h at 37°C and mounted with anti-fade medium containing DAPI (Solarbio). FISH assays were performed using a Fluorescence In Situ Hybridization Kit following the manufacturer's instructions. Confocal microscopy was performed with a Leica TCS SP5II scanning confocal microscope (Leica).

Immunohistochemical staining

Tissue slides were deparaffinized, rehydrated and washed. Antigen retrieval was performed by heating the slides in citrate buffer (pH 6.0). The sections were then incubated at 4°C overnight with primary antibodies. After that, the slides were incubated with a horseradish peroxidase-conjugated secondary antibody for 1 h, followed by incubation with 3,3'-diaminobenzidine (DAB) and H_2O_2 at RT. Visualization and imaging were performed using a Leica DM4000B microscope (Leica). The tissues were scored by counting the number of cells with positive staining in 10 separate fields at 400× magnification.

Cell Counting Kit-8 and colony formation assays

Cells with stable knockdown of circNOLC1 were plated into 96-well plates (1×10^3 cells/well) for Cell Counting Kit-8 (CCK-8) assays or 6-well plates (2×10^3 cells/well) for colony formation assays. CCK-8 solution (Dojindo) was added to each well at 0, 24, 48, and 72 hours. After incubation for 3 h, the optical density (OD) at 450 nm was measured with a Multiskan FC Microplate Photometer (Thermo Scientific). Colony formation assays were conducted for 14 days, and colonies were fixed in methanol for 15 minutes and then stained with 0.5% crystal violet (5 mg/ml) for another 30 minutes at RT. Colonies were counted with ImageJ software (NIH Image).

Cell migration assays

Cells with stable knockdown of circNOLC1 were seeded into 12-well plates and grown to 100% confluence. A scratch wound was made by using a pipette tip, and images of the same location were acquired using phase contrast microscopy (Olympus) at 0 h and 24 h. The percentage of healed area was measured as a ratio of the occupied area to the total area. For Transwell assays, 2×10^4 CRC cells in 200 μl of serum-free RPMI 1640 medium were seeded in the upper chambers (8 μm pore size, Corning Costar). The lower chambers were filled with 600 μl of complete medium. After the cells were incubated at 37°C, the cells that migrated to the bottom surface of the filter membrane were stained with 0.5% crystal violet (Sigma-Aldrich) in methanol for 45 min. Images were acquired, and the cells were counted with ImageJ software (NIH Image).

CRC organoid model

CRC tissue was minced and incubated in Advanced DMEM/F-12 medium (Gibco) and centrifuged at 850 rpm for 7 min at 4 °C. Human CRC patient-derived organoids (PDO) were established according to the manufacturer's instructions (Cat#K102, Accurate International). Briefly, organoid digestion solution I, II were added to the tissue sediment and digest at 37 ℃ for 2 hours or 10 min, respectively. After digestion is completed, centrifuge at 1500 rpm for 5 minutes. The sample was resuspended Advanced DMEM/F-12 and filtered using a 100 µm cell filter. Finally, the sample was solidified in 100% Matrigel (8 mg/mL, Corning) and cultured in Colon Cancer Organ Culture Medium (Cat#M102, Accurate International) and incubated in 5% CO2 at 37°C. Organoids were passaged approximately two weeks and passage number 3 were used in further study. Matrigel-embedded organoids were incubated with 100 µl plasma and 50 µl thrombin (20 IU/ml, Siemens), followed by overnight fix in 4% formaldehyde, then sectioned for H&E staining.

Co-culture experiment

HCT116 cells were seeded into the upper chamber (0.4 µm pore size, Corning Costar) and L-02 or BEAS-2B were seeded into the lower chamber. These cells were co-cultured for 24-48 hours and the medium is collected for cytokines and chemokines ELISA assay (Elabscience).

Luciferase reporter assay

The full-length circNOLC1 and c-Met 3'-UTRs were amplified by RT–PCR and cloned into the pmirGLO Dual-Luciferase miRNA Target Expression Vector (Promega). pmirGLO-circNOLC1 or pmirGLO-c-Met was cotransfected with miR-212-5p mimics or miR-NC into HEK-293T cells via Lipofectamine-mediated gene transfer. Cells were harvested and lysed 48 h after transfection. Firefly luciferase activity was normalized to Renilla luciferase activity for each sample.

RNA sequencing

Total RNA from HCT116 cells with stable knockdown of circNOLC1 and control cells was extracted using TRIzol. RNA purity was checked using a K5500 spectrophotometer (Kaiao).

RNA integrity and concentration were assessed using an RNA Nano 6000 Assay Kit of the Bioanalyzer 2100 system (Agilent Technologies). Transcriptome deep-sequencing was performed by ANNOROAD Gene Technology (Beijing, China). Briefly, a total amount of 2 μg RNA per sample was used as input material for RNA sample preparation. Sequencing libraries were generated using NEBNext Ultra RNA Library Prep Kit for Illumina (NEB) according to the manufacturer's recommendations, and index codes were added to attribute sequences to each sample. Subsequently, RNA concentration of library was diluted to 1 ng/μl. The insert size was assessed using the Agilent Bioanalyzer 2100 system (Agilent Technologies), and qualified insert size was accurately quantified using a StepOnePlus Real-Time PCR System (Library valid concentration >10 nM). Clustering of the index-coded samples was performed on a cBot cluster generation system using a HiSeq PE Cluster Kit v4-cBot-HS (Illumina). After that, the libraries were sequenced on the Illumina NovaSeq 6000 platform, and 150 bp paired-end reads were generated. Finally, Bowtie software was used to build the reference genome index, and HISAT2 software was utilized to align clean reads to the reference genome. Differential analysis of the RNA-seq data was performed using DESeq2. Genes with [log2 (fold change)] ≥1 and q value ≤0.05 were considered differentially expressed genes. Gene ontology analysis was carried out with the DAVID tool.

Glucose consumption, lactate production, and G6PD enzyme activity assays

Cells were seeded into culture plates and cultured overnight. For glucose consumption and lactate production assays, cells were collected by centrifugation and suspended in distilled water. Suspensions were sonicated in an ice-water bath and centrifuged to obtain the supernatants. Glucose levels in the supernatants were determined using a Glucose Assay Kit (Solarbio). Lactate levels were determined using a Lactate Assay Kit (KeyGen). G6PD activity was determined using a Glucose-6-Phosphate Dehydrogenase Activity Assay Kit (Solarbio).

Quantification of the intracellular NADPH level and NADP+/NADPH ratio

The NADPH level was measured using an NADPH Enzyme-Linked Immunosorbent Assay Kit (JiNing Biotech) according to the manufacturer's instructions. Briefly, cells were seeded

into 10 cm dishes and cultured overnight. Then cells were lysed in extraction buffer and centrifuged. The microtiter plates coated with antibody to NADPH were incubated with the supernatant, followed by the addition of horseradish peroxidase (HRP)-conjugated antibody. Color development was achieved by adding 3,3',5,5'-tetramethyl-benzidine (TMB) as a substrate to each well. Sulfuric acid was added to stop the reaction. The OD values at 450 nm were measured using a Multiskan FC Microplate Photometer (Thermo Scientific). $NADP^+$/NADPH ratios in cell lines were measured by an $NADP^+$/NADPH Assay Kit (Beyotime) according to the manufacturer's recommendations. According to the NADPH standards, the concentration of total NADP or NADPH can be expressed in pmol per 10^6 cells. The $NADP^+$/NADPH ratio was calculated as [($NADP_{total}$)-(NADPH)]/(NADPH).

Reactive oxygen species (ROS) level

To determine the level of ROS production, cells were stained with 2',7'-dichlorodihydrofluorescein diacetate (DCFH-DA) using an ROS Assay Kit (Beyotime). Briefly, cells were washed with medium and incubated with DCFH-DA (10 μM) in the dark at 37°C for 20 min. DCFH-DA was deacetylated intracellularly by nonspecific esterase, which was further oxidized by ROS to the fluorescent compound 2',7'-dichlorofluorescein (DCF). DCF fluorescence was detected using fluorescence microscopy (Olympus). The results are expressed as the mean fluorescence intensity per cell.

5-Ethynyl-2'-deoxyuridine (EdU) incorporation assay

DNA synthesis was evaluated using a BeyoClick EdU Cell Proliferation Kit (Beyotime). Cells were seeded into 6-well plates and incubated with EdU solution (10 μM) for 2 h at 37°C. Next, the cells were fixed with 4% paraformaldehyde and permeabilized with 0.3% Triton X-100. EdU was labeled with Alexa Fluor® 555 azide via a copper-catalyzed click reaction. Nuclei were stained with Hoechst 33342. Images were acquired using fluorescence microscopy (Olympus), and EdU-positive cells were counted.

Determination of Lipid Synthesis

Cells were seeded in 6-well plates and stained with an Oil Red O (ORO) Staining Kit (Solarbio). Cells were fixed with ORO fixative, dipped in 60% isopropanol for 5 min, and

stained with the ORO staining reagent for 20 min. Nuclei were stained with Mayer's hematoxylin staining solution for 2 min. Images were acquired using microscopy (Olympus), and positive-stained cells were counted.

Glucose uptake assay

Cells were seeded into 12-well plates and cultured overnight. The media were collected and diluted 1:1000 (RPMI 1640 medium) or 1:2000 (DMEM) with distilled water. The amount of glucose in the media was then determined using an Amplex Red Glucose Assay Kit (Invitrogen) according to the manufacturer's instructions. Glucose uptake was determined by subtracting the amount of glucose in each sample from the total amount of glucose in medium without cells.

circRNA in vivo precipitation (circRIP)

Biotin-labeled circNOLC1 and control probes were synthesized by Sangon Biotech. Cells were seeded into a 10 cm dish and transfected with the specific biotin-labeled circNOLC1 or control probe at a final concentration of 200 nM. Cells were harvested after transfection for 24 h, crosslinked with 1% formaldehyde for 10 min, lysed in co-IP buffer, and sonicated. After centrifugation, 5% of the supernatant was retained as input, and the remaining was incubated with M280 streptavidin Dynabeads (Invitrogen) overnight at 4°C. After that, the probe-dynabeads-circRNA mixture was washed and incubated with 200 μl of lysis buffer and proteinase K to reverse formaldehyde crosslinking. Subsequently, TRIzol was added to the mixture for RNA extraction and detection.

Bioinformatics prediction

CatRAPID, an algorithm used to estimate the binding propensity of RNA-protein pairs, was used to predict the binding site between circNOLC1 and AZGP1 by combining the secondary structure, hydrogen bonding, and van der Waals contributions. The databases starbase, circbank and TargetScan were applied to analyze the potential miRNA targeting sites within circRNA and c-Met 3'-UTR.

Supplementary Tables

Supplementary Table S1. The list of differential circRNA with same expression trends of their parental genes in TCGA database.

circRNA ID	Origin	Strand	T *vs* N	*P* value	Parental gene	Deregulated
hsa_circ_0000257	exonic	+	3.12	0.0246	NOLC1	Up
-	exonic	+	2.60	0.0447	PCCB	Up
-	exonic	+	2.37	0.0231	CCNF	Up
hsa_circ_0088494	exonic	+	2.23	0.0323	NEK6	Up
hsa_circ_0089863	exonic	+	2.06	0.0046	SHROOM2	Up
hsa_circ_0003738	exonic	+	2.01	0.0041	LRRC16A	Up
hsa_circ_0069922	exonic	+	0.47	0.0048	SLC4A4	Down
hsa_circ_0005856	exonic	-	0.47	0.0365	ZZEF1	Down
hsa_circ_0069715	exonic	+	0.47	0.0086	DCUN1D4	Down
hsa_circ_0001722	exonic	+	0.47	0.0304	CDK14	Down
-	exonic	+	0.44	0.0088	PLCG2	Down
hsa_circ_0004702	exonic	-	0.44	0.0224	PLEKHM1P	Down
hsa_circ_0016121	exonic	+	0.44	0.0039	SOX13	Down
hsa_circ_0003611	exonic	-	0.36	0.0021	LPAR1	Down
hsa_circ_0016771	exonic	+	0.36	0.0085	OBSCN	Down
hsa_circ_0009024	exonic	+	0.36	0.0187	TXLNGY	Down
hsa_circ_0038343	exonic	+	0.35	0.0371	C16orf62	Down
hsa_circ_0042488	exonic	+	0.32	0.0127	WSB1	Down
-	exonic	-	0.32	0.0384	EIF4E3	Down
hsa_circ_0004767	exonic	-	0.31	0.0064	ZZEF1	Down
hsa_circ_0035796	exonic	-	0.29	0.0039	HERC1	Down
hsa_circ_0040609	exonic	+	0.29	0.0119	PLCG2	Down
hsa_circ_0019225	exonic	+	0.28	0.0021	PLCE1	Down
hsa_circ_0073244	exonic	-	0.27	0.0017	EDIL3	Down
hsa_circ_0026457	exonic	-	0.26	0.0008	KRT5	Down
hsa_circ_0001212	exonic	-	0.26	0.0122	GUSBP11	Down
hsa_circ_0008267	exonic	+	0.19	0.0077	LINC00969	Down

Supplementary Table S2. The 9 circRNAs whose parental genes are reported to have association with CRC progression.

**circRNA**	**Parental gene**	**Function of parental gene**
circNOLC1	NOLC1	Function as P53 and MYC target gene in CRC.
circCCNF	CCNF	Resistant to the chemotherapeutic agent 5-fluorouracil in CRC.
circNEK6	NEK6	Associated with the development of adenomatous colorectal polyps and CRC.
circSHROOM2	SHROOM2	Influence the occurrence and risk of CRC.
circSLC4A4	SLC4A4	Potential biomarker for the diagnosis and prognosis of CRC.
circSOX13	SOX13	Promotes metastasis of CRC.
circLPAR1	LPAR1	Promotes metastasis of CRC.
circPLCE1	PLCE1	Influence the occurrence and risk of CRC.
circKRT5	KRT5	Potential biomarker for the diagnosis of CRC.

Supplementary Table S3. The CPC2 predicts the protein-coding potential of circNOLC1.

Gene	circRNA ID	RNA Size	ORF Size	Peptide length (aa)	Fickett score[a]	Coding probability
NOLC1	hsa_circ_0000257	487	351	116	1.213	0.28863147863328

ORF: Open Reading Frame

[a]Fickett Score is the statistic that measures the computational bias between coding and non-coding sequences based on a periodicity of three (every three nucleotides encode one amino acid).

Supplementary Table S4. Proteins identified in whole eluted proteins by CHIRP-MS may interact with circNOLC1.

**Protein**	**Proteins Number**	**Unique peptides**	**Protein**	**Proteins Number**	**Unique peptides**
KRT10	40	25	HNRNPF	5	2
KRT9	3	23	TUBB	61	2
KRT2	27	20	ATP5B	6	2
ALB	17	12	HSP90AA1	15	2
KRT16	8	11	HSP90AB1	16	2
HNRNPH1	28	10	EEF1A1	38	2
DSP	9	9	KRT78	5	2
HNRNPK	11	8	DCD	2	2
HNRNPA3	9	8	KRT5	4	2
KRT14	27	7	AZGP1	3	2
PKM	26	7	DSG1	1	2
HNRNPA1	21	7	KRT6A	13	2
ANXA2	29	6	HSPE1	5	1
ENO1	27	5	TMEM33	4	1
H4	4	5	RPLP2	3	1
TBA1C	40	5	UBC	39	1
RBMX	15	5	S100A7	1	1
KRT17	9	5	H2B1C	22	1
HSPA8	24	4	FLG2	2	1
HSPD1	14	3	SPTBN2	2	1
HSPB1	5	3	ATP6	1025	1
H2A1H	34	3	RPL14	7	1
GAPDH	9	3	KRT6C	4	1
JUP	4	3	PIP	1	1
FABP5	3	3	HRNR	2	1

Supplementary Table S5. Proteins identified in whole eluted proteins by MS may specifically interact with circNOLC1 compared to negative control.

**Protein**	**NC Peptide**	**circNOLC1 Peptide**	**Fold change**	**Annotation**
FABP5	0	4	2.28	Fatty acid binding protein 5
KRT16	5	23	1.95	Keratin, type I cytoskeletal 16
KRT6A	8	31	1.78	Keratin, type II cytoskeletal 6A
AZGP1	0	2	1.55	Alpha-2-glycoprotein 1, zinc-binding
DSG1	0	2	1.55	Desmoglein-1
DSP	3	10	1.42	Desmoplakin
KRT17	2	6	1.18	Keratin, type I cytoskeletal 17
KRT5	18	39	1.03	Keratin, type II cytoskeletal 5

Supplementary Table S6. The target miRNAs of circNOLC1 predicted by starbase and circbank databases.

miRNA	starbase (binding sites number)	circbank (binding sites number)		Expression status	
	CLIP-Seq	miRanda	TargetScan	TCGA	GSE56350
miR-212-5p	3	1	4	Down-regulated	Down-regulated
miR-665	2	1	2	No significant	Not available
miR-650	2	1	4	Not available	No significant
miR-3142	1	1	2	Not available	Not available
miR-330-5p	1	1	2	Down-regulated	Not available
miR-326	1	1	2	Down-regulated	No significant
miR-3184-5p	1	1	2	Not available	Not available

Supplementary Table S7. Potential YY1 binding site on NOLC1 promoter.

Gene	Score	Start	End	Strand	Predicted site sequence
YY1	7.079	216	227	+	GAACATGGTCCA
YY1	5.985	662	673	+	GGAGATGGAGTC
YY1	8.191	1713	1718	-	ACCATC
YY1	7.219	1791	1796	+	GCCATG

Supplementary Table S8. The information of the patients with liver metastases from CRC.

No.	Sex	Age (years)	Tumor location	Histological grading	Liver metastasis	TNM stage
CRLM1	Female	38	Colon	Moderately differentiated adenocarcinoma	Yes	IV
CRLM2	Male	55	Colon	Moderately differentiated adenocarcinoma	Yes	IV
CRLM3	Female	62	Colon	Moderately and poorly differentiated adenocarcinoma	Yes	IV
CRLM4	Male	62	Colon	Moderately differentiated adenocarcinoma	Yes	IV
CRLM5	Female	57	Colon	Mucinous/Moderately differentiated adenocarcinoma	Yes	IV
CRLM6	Female	32	Rectum	Moderately differentiated adenocarcinoma	Yes	IV
CRLM7	Male	64	Colon	Moderately differentiated adenocarcinoma	Yes	IV
CRLM8	Male	60	Colon	Poorly differentiated adenocarcinoma	Yes	IV
CRLM9	Female	63	Colon	Moderately and poorly differentiated adenocarcinoma	Yes	IV
CRLM10	Male	66	Colon	Moderately and poorly differentiated adenocarcinoma	Yes	IV
CRLM11	Male	62	Colon	Moderately differentiated adenocarcinoma	Yes	IV
CRLM12	Female	61	Colon	Mucinous/Poorly differentiated adenocarcinoma	Yes	IV
CRLM13	Female	57	Colon	Moderately differentiated adenocarcinoma	Yes	IV
CRLM14	Male	72	Colon	Moderately differentiated adenocarcinoma	Yes	IV
CRLM15	Female	52	Rectum	Moderately differentiated adenocarcinoma	Yes	IV
CRLM16	Female	55	Colon	Moderately differentiated adenocarcinoma	Yes	IV
CRLM17	Male	59	Rectum	Moderately differentiated adenocarcinoma	Yes	IV
CRLM18	Male	63	Rectum	Moderately differentiated adenocarcinoma	Yes	IV
CRLM19	Male	52	Rectum	Moderately differentiated adenocarcinoma	Yes	IV
CRLM20	Female	64	Colon	Moderately differentiated adenocarcinoma	Yes	IV

Supplementary Table S9. The antibodies used in this study.

Name	Host	Product code	Company
mTOR (for WB)	Rabbit pAb	WL02477	Wanleibio
SREBP1(for WB, IHC)	Rabbit pAb	K106528P	Solarbio
G6PD (for WB, IHC)	Rabbit pAb	K002820P	Solarbio
AZGP1 (for IP)	Rabbit pAb	Ab180574	Abcam
AZGP1(for WB, IF)	Mouse mAb	66178-1-Ig	Proteintech
DSG1	Rabbit pAb	24587-1-AP	Proteintech
FABP5	Rabbit pAb	12348-1-AP	Proteintech
KRT16	Rabbit pAb	17265-1-AP	Proteintech
KRT17	Rabbit pAb	17516-1-AP	Proteintech
DSP	Rabbit pAb	25318-1-AP	Proteintech
KRT5	Mouse mAb	66727-1-Ig	Proteintech
KRT6A	Rabbit pAb	10590-1-AP	Proteintech
AGO2	Rabbit mAb	ab186733	Abcam
c-Met (for WB, IHC)	Mouse mAb	sc-514148	Santa Cruz
NOLC1	Rabbit pAb	11815-1-AP	Proteintech
YY1(for WB, IP)	Rabbit pAb	22156-1-AP	Proteintech
β-actin (for WB)	Mouse mAb	TA-09	ZSGB-Bio

Supplementary Table S10. Primers, probes and siRNA sequences used in this study.

Gene name	Sequence (5' to 3') Forward	Sequence (5' to 3') Reverse	Amplified size
**Quantitative RT-PCR primers**			
circNOLC1	TCCGAGGATGAGCCACCA	ACTTTCGCTCTGGGACCTT	167
circNOLC1 (Linear)	CGAAAGTTACAGGCAAATGG	GGACTCTTCACTGCTGCTACTCT	201
NOLC1 mRNA	GCAGCCAAAGCTCCTCCTAA	GCTTCTGGTTCTTTGGTGGC	88
NOLC1 pre-mRNA	AGGATGAGCCACCAAAGAACC	CAGCCTCTTGGGAACAGAAAC	107
G6PD	ACCGCATCGACCACTACCT	TGGGGCCGAAGATCCTGTT	81
SREBF1	TTGCCGACCCTGGTGAGT	AATGGCGTTGTGGGCTGT	153
mTOR	ATCCAGACCCTGACCCAAAC	TCCACCCACTTCCTCATCTC	100
GLRX	GGGAAGGTGGTTGTGTTCAT	TAGTTTGGTTGGTGGCTGTG	127
PGD	ATGCCAGGAGGGAACAAAG	GTTCTCCGGTTCCCACTTTT	82
PGLS	CAGACTGCCGATCCCAGAAAG	CCCTTGGAATGCCTGTCTCA	106
PGM2L1	TGGTTGGGTATGACACTCGG	GTCTTCCTTGCGGTTGTGAGA	209
RPE	CAGGAGCCAATCAGTACACCT	CAAGGCCAACCTGCAATGG	150
RPIA	TCTGAACCTCGTCTGTATTCCC	ACTGAGGGTCAAGCCATACTG	73
SLC37A4	AGGTAGCTCCTACATGAGTGC	GGGTTCCCGTAGTTGGACAG	114
TALDO1	CAGCACAGATGCCCGCTTA	CGGCCCGGAATCTTCTTTAGTA	151
TKT	TCCACACCATGCGCTACAAG	CAAGTCGGAGCTGATCTTCCT	164
YY1	AAGAAGTGGGAGCAGAAGCA	CAACCACTGTCTCATGGTCAA	109
c-Met	CAGATGTGTGGTCCTTTG	ATTCGGGTTGTAGGAGTCT	129
miR-212-5p	GCTTACGCTTCGAGCCCAC	GACACCACGGCCCACTCTGCA	
HuR	AGAGCGATCAACACGCTGAA	TAAACGCAACCCCTCTGGAC	229
AUF1	GCGTGGGTTCTGCTTTATTACC	TTGCTGATATTGTTCCTTCGACA	130
ChIRP-circNOLC1	CCGAGGATGAGCCACCAAAG	CATCCTGCTGTGTCTGGTTTG	
GAPDH	CCTCAAGATCATCAGCAAT	CCATCCACAGTCTTCTGGGT	141
U6	CTCGCTTCGGCAGCACA	AACGCTTCACGAATTTGCGT	
**ChIP primers**			
S1	CCTGGGTATTGGAGGCAGTG	TACAAGATCTCCCAACCGCC	85
S2	ACGGATAACCCGAAGGATCT	CTTCACTGCCTCCAATACCC	95
S3	TGCATGTGGGTAGAACAGGA	TGCAGTAAGCCAAAATCGTG	147
S4	CAACTGCTGTTGTGCGTCTT	GTGGGATTTTTGCACCTGAC	110
Ctrl S	GGGAAAAGTTGGGCTGCTTAC	AGATCCGATTCTACGCTTCCAA	102

**sgRNA sequence**		
sgRNA-G6PD	CACCGACGAAGCGCAGGTAACCGGC	AAACGCCGGTTACCTGCGCTTCGTC
sgRNA-AluSq-1	CACCGCAACTTTAGAGCAGCCATCG	AAACCGATGGCTGCTCTAAAGTTGC
sgRNA-AluSq-2	CACCGATGGCTGGCATTCTCCTCGA	AAACTCGAGGAGAATGCCAGCCATC
sgRNA-AluSq-3	CACCGTAAGTTCTCTGCTCATAGGA	AAACTCCTATGAGCAGAGAACTTAC
sgRNA-AluSq-4	CACCGTTCTCTGCTCATAGGAAGG	AAACCCTTCCTATGAGCAGAGAAC
**Biotin labeled probes**	**Sequence (5' to 3')**	
Sense-circNOLC1	ATTGGCATCCTGCTGTGTCTGGTTTGGGAGGGGCTTTA	
Antisense-circNOLC1	TAACCGTAGGACGACACAGACCAAACCCTCCCCCAAAT	
circNOLC1 91-150bp	AAAUGGACCAGUGGCUAAGAAAGCUAAGAAGAAGG CCUCAUCCAGUGACAGUGAGGACAG	
circNOLC1 151-300bp	CAGCGAGGAGGAGGAGGAAGTTCAAGGGCCTCCAGCAAA GAAGGCTGCTGTACCTGCCAAGCGAGTCGGTCTGCCTCCT GGGAAGGCTGCAGCCAAAGCATCAGAGAGTAGCAGCAGT GAAGAGTCCAGTGATGATGATGATGA GGAGGAC	
miR-212-5p	ACCUUGGCUCUAGACUGCUUACU	
miRNA-NC	AGUAAGCAGUCUAGAGCCAAGGU	
**ChIRP probes**	**Sequence (5' to 3')**	
circNOLC1-P1	GACTTGAGCCAGAAGCTATA	
circNOLC1-P2	TGCTACTCTCTGATGCTTTG	
circNOLC1-P3	CTGTCACAGGTGTTATCTTT	
circNOLC1-P4	ATCCTGCTGTGTCTGGTTTGGGAG	
Control-P1	AATACGCGTCACTACCGTTG	
Control-P2	TCGCTTTGGCGAACTTATTG	
Control-P3	ACCATTGGCTATTTTAGGTG	
Control-P4	GCTTTTTAGGTACAGTCTTC	
**Fluorescein labeled ISH probes**	**Sequence (5' to 3')**	
5'-Cy3-circNOLC1	CAUCCUUGCUGUGUCUGGUUUGGGAGGG	
5'-FAM-miR-212-5p	AGTAAGCAGTCTAGAGCCAAGGT	
Cy3-NC	GTGTAACACGTCTATACGCCCA	
FAM-NC	GTGTAACACGTCTATACGCCCA	
**shRNA or siRNA sequence**	**Sequence (5' to 3')**	
circNOLC1 shRNA#1	CCAAACCAGACACAGCAGGAT	
circNOLC1 shRNA#2	ACCAGACACAGCAGGATGCCA	
Scramble shRNA	TTCTCCGAACGTGTCACGT	

SREBF1 siRNA	CGGAGAAGCUGCCUAUCAATT
AZGP1 siRNA	GUUGUGAGAUCGAGAAUAATT
YY1 siRNA	GCACAAAGATGTTCAGGGA
HuR siRNA	AAGAGGCAATTACCAGTTTCA
AUF1 siRNA	AAGAUCCUAUCACAGGGCGAT
Scramble siRNA	UUAUUCUCGAUCUCACAACCA
miRNA-NC	UUGUACUACACAAAAGUACUG

Supplementary Figures and Figure legends

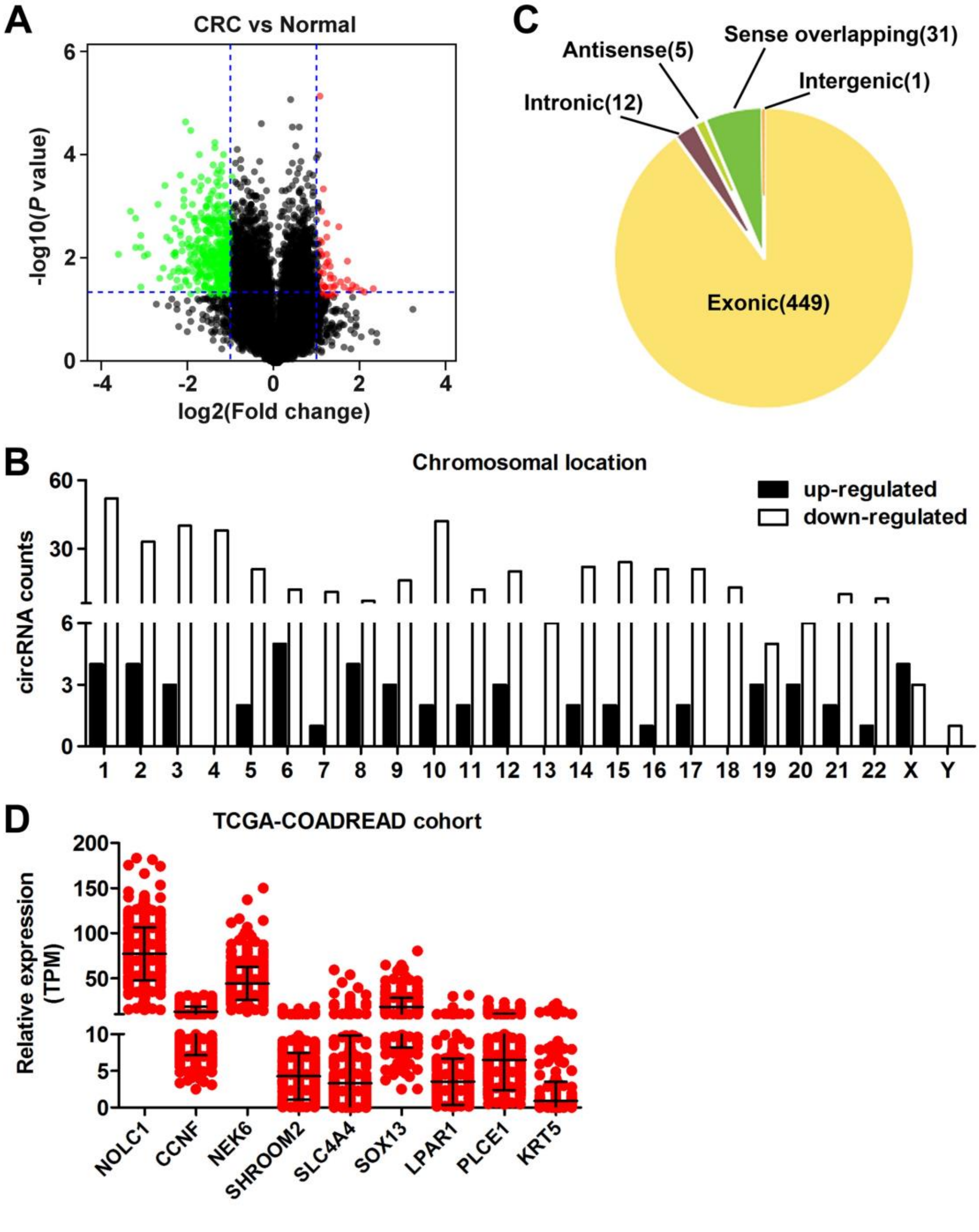

Figure S1. Related to Figure 1. (A) Volcano plots of differential circRNA expression between CRC tissues and paired adjacent normal tissues. (B) Chromosomal distribution of upregulated and downregulated circRNAs in human colorectal tissues. (C) Genomic origin of the circRNAs identified in human colorectal tissues. (D) Differential expression of host gene

transcripts corresponding to the top 9 circRNAs in the TCGA-COADREAD cohort. *$p < 0.05$.

CRC, colorectal cancer; TCGA, The Cancer Genome Atlas.

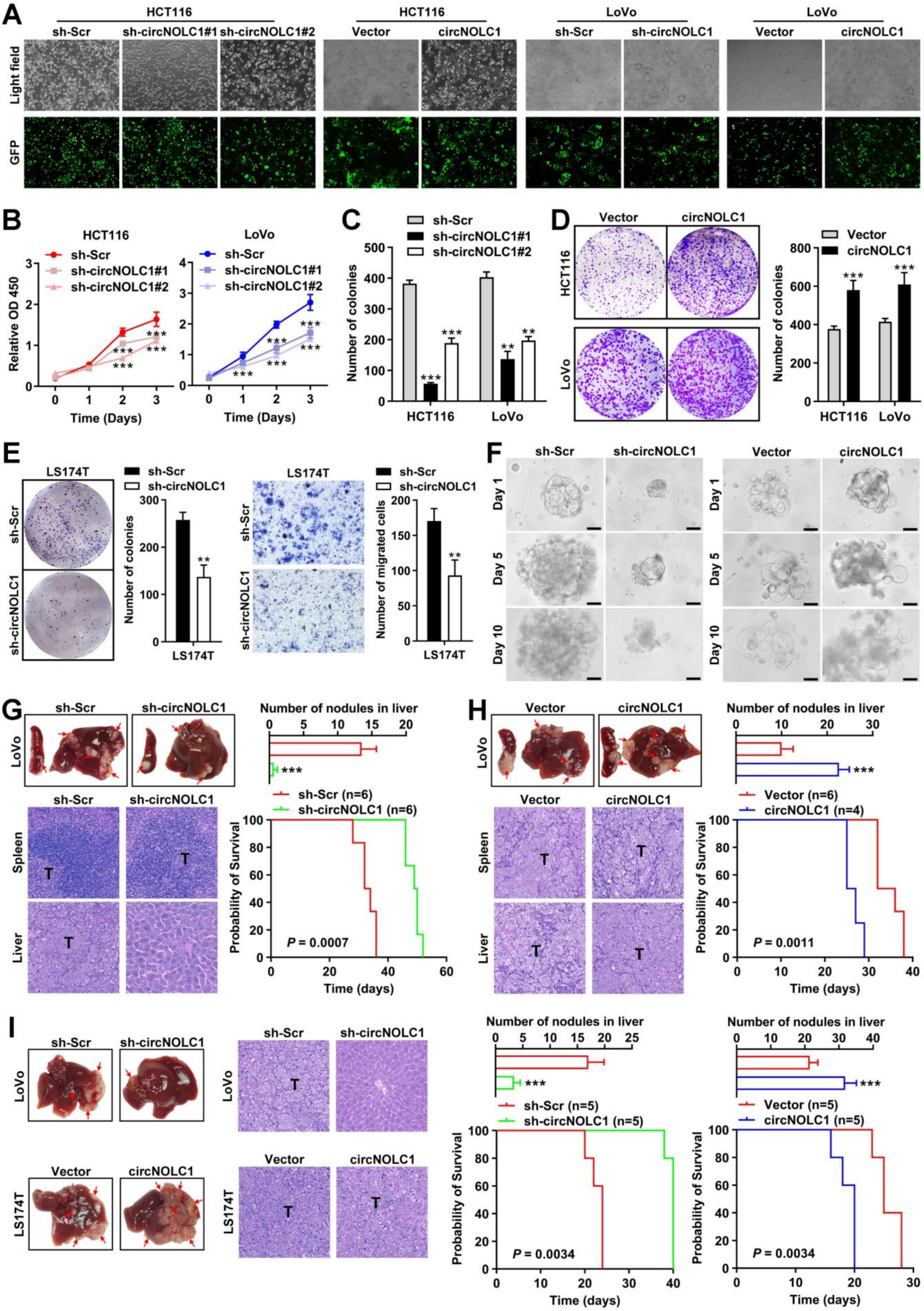

A
HCT116
sh-Scr
sh-circNOLC1#1
sh-circNOLC1#2
HCT116
Vector
circNOLC1
LoVo
sh-Scr
sh-circNOLC1
LoVo
Vector
circNOLC1
Light field
GFP
B
HCT116
sh-Scr
sh-circNOLC1#1
sh-circNOLC1#2
Relative OD 450
Time (Days)
LoVo
sh-Scr
sh-circNOLC1#1
sh-circNOLC1#2
Time (Days)
C
sh-Scr
sh-circNOLC1#1
sh-circNOLC1#2
Number of colonies
HCT116
LoVo
D
Vector
circNOLC1
HCT116
LoVo
Vector
circNOLC1
Number of colonies
HCT116
LoVo
E
LS174T
sh-Scr
sh-circNOLC1
sh-Scr
sh-circNOLC1
Number of colonies
LS174T
LS174T
sh-Scr
sh-circNOLC1
sh-Scr
sh-circNOLC1
Number of migrated cells
LS174T
F
sh-Scr
sh-circNOLC1
Vector
circNOLC1
Day 1
Day 5
Day 10
G
sh-Scr
sh-circNOLC1
LoVo
Number of nodules in liver
sh-Scr (n=6)
sh-circNOLC1 (n=6)
Spleen
Liver
Probability of Survival
P = 0.0007
Time (days)
H
Vector
circNOLC1
LoVo
Number of nodules in liver
Vector (n=6)
circNOLC1 (n=4)
Spleen
Liver
Probability of Survival
P = 0.0011
Time (days)
I
sh-Scr
sh-circNOLC1
LoVo
Vector
circNOLC1
LS174T
Number of nodules in liver
sh-Scr (n=5)
sh-circNOLC1 (n=5)
Probability of Survival
P = 0.0034
Time (days)
Number of nodules in liver
Vector (n=5)
circNOLC1 (n=5)
Probability of Survival
P = 0.0034
Time (days)

Figure S2. Related to Figure 2. (A) The lentiviral transduction efficiency in HCT116 and LoVo cells. Because the vector contains a GFP fragment, the cells emit green fluorescence when transduced with the lentivirus. (B) The effect of circNOLC1 knockdown on the proliferation ability of CRC cells was evaluated by CCK-8 assays. (C, D) The effect of circNOLC1 knockdown (C) or overexpression (D) on the proliferation ability of human CRC cells was evaluated by colony formation assays. (E) The effect of circNOLC1 knockdown on the proliferation and migration ability of LS174T cells was evaluated by colony formation and Transwell assays, respectively. (F) Representative images of Matrigel-suspended organoids transduced with lentiviral vectors containing circNOLC1 knockdown or overexpression cultured for 1, 5, 10 days. Scale bar: 50 μm. (G, H) The spleen and liver were examined at the gross anatomical level for tumors and metastases after intra-splenic injection with circNOLC1 knockdown (G) or overexpressed (H) LoVo cells. The red arrows indicate tumor foci. Representative H&E staining showing primary (upper) and metastatic (lower) nodules. T, tumor. The number of metastatic nodules in the livers. Kaplan–Meier survival curves for mice implanted with circNOLC1 knockdown or overexpressed LoVo cells. (I) The liver was examined at the gross anatomical level for tumors and metastases after liver capsule (Glisson's capsule) injection with circNOLC1 knockdown LoVo or overexpressed LS174T cells. The red arrows indicate tumor foci. Representative H&E staining showing metastatic nodules. T, tumor. The number of metastatic nodules in the livers. Kaplan–Meier survival curves for mice implanted with circNOLC1 knockdown LoVo or overexpressed LS174T cells. **$p < 0.01$, ***$p < 0.001$. GFP, green fluorescent protein; CRC, colorectal cancer; CCK-8, Cell Counting Kit-8.

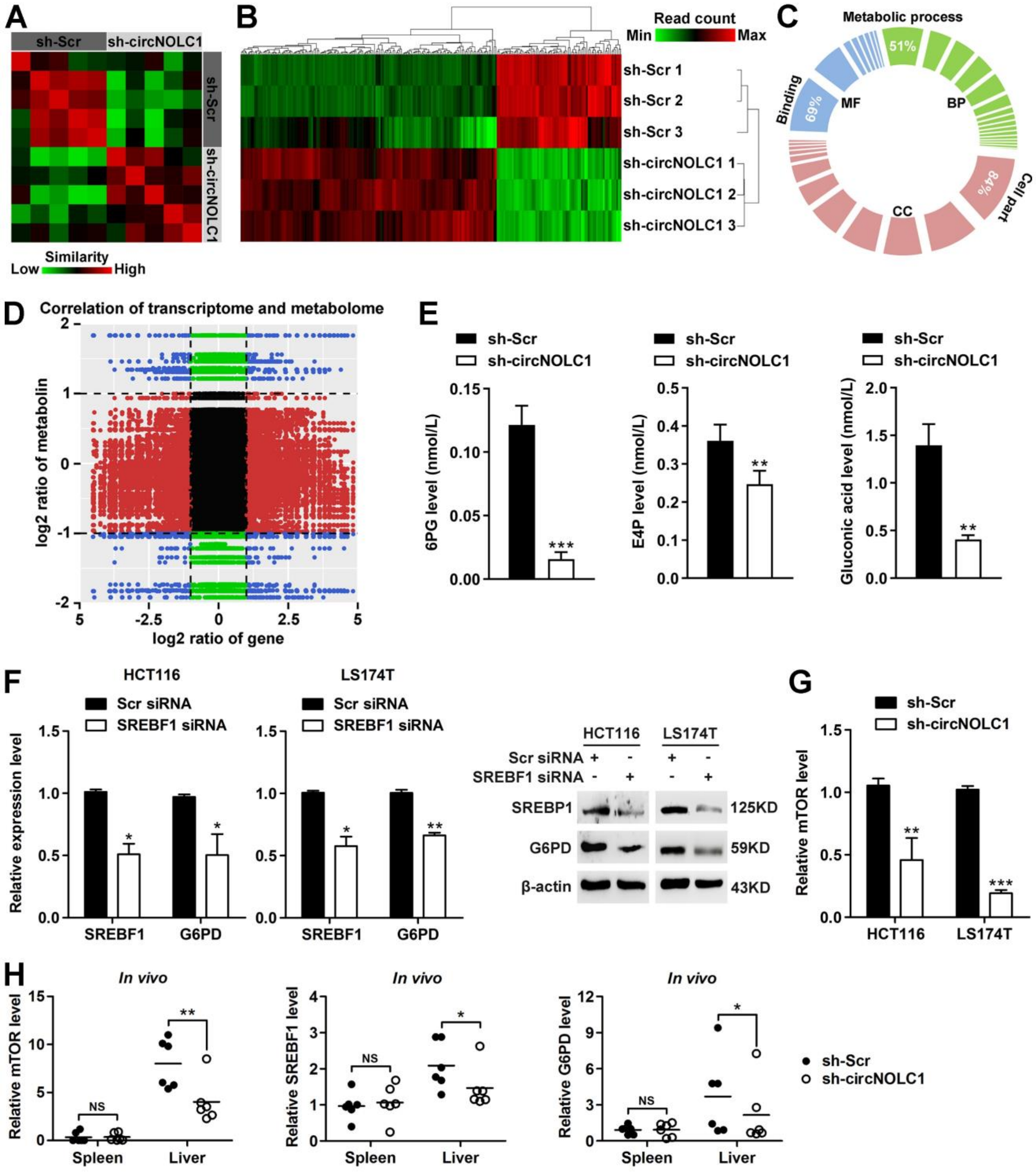

Figure S3. Related to Figure 3. (A) Similarity matrix analysis of metabolite clustering in the metabolome of HCT116 cells expressing control and circNOLC1 shRNA. The similarity matrix was generated using Morpheus based on Euclidean distance analysis to evaluate the metabolomic differences between samples. (B) Heatmap of RNA-seq analysis from HCT116 cells expressing control or circNOLC1 shRNA. (C) Functional annotation clustering of genes

regulated by circNOLC1 in HCT116 cells is shown. Significantly enriched groups designated by the gene ontology term are ranked based on the group enrichment scores. Red indicates cellular component (CC) terms; green, biological process (BP) terms; and blue, molecular function (MF) terms. (D) Transcriptome–metabolome correlation. The Pearson correlation coefficients of genes and metabolites greater than 0.8 in each differential subgroup were divided into nine quadrants from left to right and from top to bottom. Quadrants 3 and 7 represent positive correlation between genes and metabolites; quadrants 1 and 9 represent negative correlation. (E) Intermediate metabolites of the pentose phosphate pathway were extracted from HCT116 cells expressing control shRNA or circNOLC1 shRNA and confirmed by mass spectrometry. (F) HCT116 and LS174T cells were transiently transfected with scramble or SREBF1 siRNA, and the isolated total RNA and lysates were applied to qPCR and western blot analysis. (G) The expression level of mTOR in circNOLC1-silenced CRC cells was determined using qPCR. (H) Stable HCT116 cells expressing control or circNOLC1 shRNA were injected into the spleen. Then total RNA of spleen and liver tissues was extracted and applied to qPCR. *$p < 0.05$, **$p < 0.01$, ***$p < 0.001$; NS, not significant. RNA-seq, RNA sequencing; siRNA, small interfering RNA; CRC, colorectal cancer; qPCR, quantitative reverse transcription PCR.

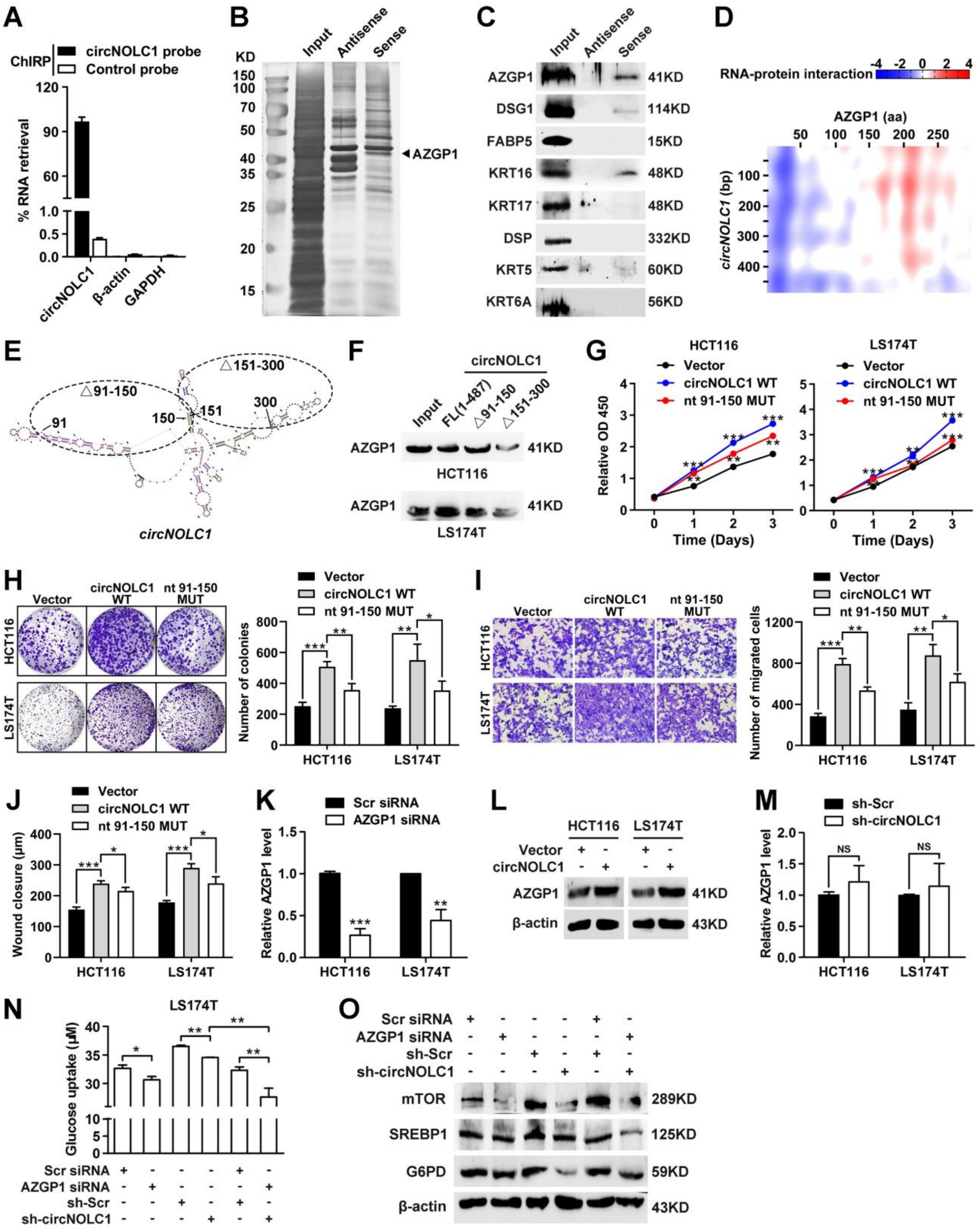

Figure S4. Related to Figure 4. (A) ChIRP enriched for circNOLC1 RNA in CRC cells. circNOLC1 probes retrieved over 95% of cellular circNOLC1 RNA and undetectable β-actin

or GAPDH. (B) Silver staining of proteins bound to the circNOLC1 sense (right lane) or antisense (middle lane) probes. The RNA pull-down assay was performed with LS174T cell lysates. A specific band (arrow) was identified as AZGP1 by mass spectrometry. (C) AZGP1, DSG1, and KRT16 bound to circNOLC1 as confirmed by western blot after RNA pull-down. (D) Prediction of the RNA-protein interaction of circNOLC1 with AZGP1 using the catRAPID algorithm. (E) Identification of matched binding sites in relation to circNOLC1 secondary structure analysis. (F) Western blot analysis of AZGP1 protein expression in RNA pull-down products with one full-length probe and two truncated probes. (G-H) The effect of nt 91-150 substitution mutation or full-length circNOLC1 overexpression on the proliferation ability of CRC cells was evaluated by CCK-8 (G) and colony formation (H) assays, respectively. (I-J) The effect of nt 91-150 substitution mutation or full-length circNOLC1 overexpression on the migration ability of CRC cells was evaluated by Transwell (I) and wound healing (J) assays, respectively. (K) Efficient knockdown of AZGP1 in CRC cells was detected by qPCR. (L) The protein level of AZGP1 in circNOLC1-overexpressing CRC cells was determined using western blot analysis. (M) The mRNA level of AZGP1 in circNOLC1-silenced CRC cells was determined using qPCR. (N) Glucose uptake level determined by Amplex Red assay in culture media from circNOLC1-and/or AZGP1-silenced LS174T cells. Total amount of glucose in the media was used for normalization. (O) Western blot analysis of HCT116 cells expressing control or circNOLC1 shRNA in combination with scramble or AZGP1 siRNA as indicated. *$p < 0.05$, **$p < 0.01$, ***$p < 0.001$; NS, not significant. ChIRP, chromatin isolation by RNA purification; MS, mass spectrometry; siRNA, small interfering RNA; CRC, colorectal cancer; qPCR, quantitative reverse transcription PCR.

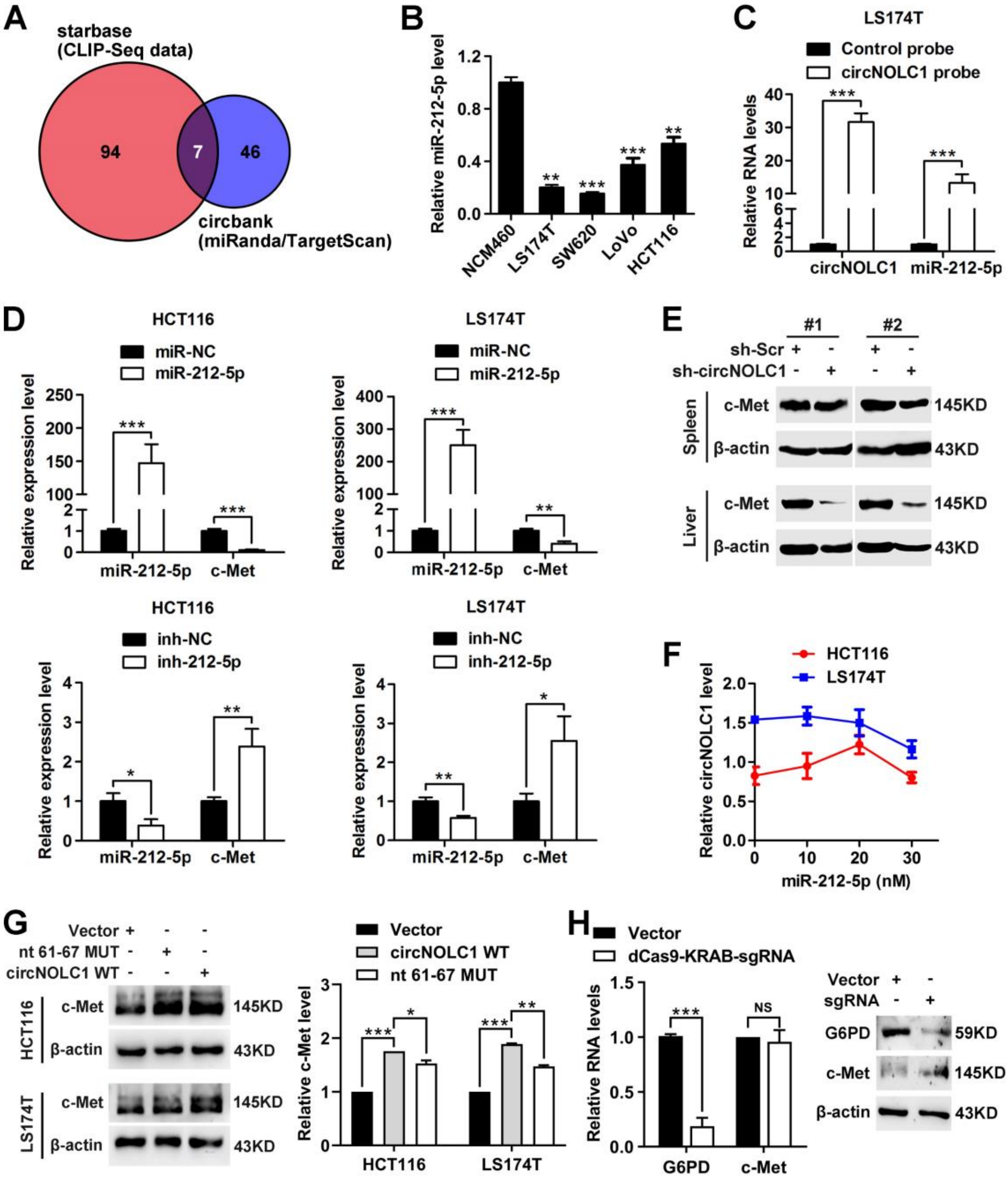

Figure S5. Related to Figure 5. (A) Two independent miRNA–circRNA interaction databases were used to screen the potential miRNAs binding to circNOLC1. (B) miR-212-5p expression in CRC cell lines was lower than that in the normal colonic epithelial cell line as determined by qPCR. (C) miR-212-5p was pulled down and enriched with a circNOLC1-specific probe and then detected by qPCR. (D) The mRNA level of c-Met was determined by qPCR in CRC cells after transfection with miR-212-5p mimics or inhibitors.

(E) Stable LS174T cells expressing control or circNOLC1 shRNA were injected into the spleen. The lysates of spleen and liver tissues were applied to western blot analysis. (F) The expression level of circNOLC1 was determined by qPCR in CRC cells after transfection with different concentrations of miR-212-5p mimics. (G) HCT116 and LS174T cells were transiently transfected with nt 61-67 substitution mutation or full-length circNOLC1, and the lysates were applied to western blot analysis. (H) G6PD and c-Met levels in HCT116 cells expressing dCas9-KRAB were detected by qPCR and western blot analysis after transfection with sgRNA targeting the G6PD. *$p < 0.05$, **$p < 0.01$, ***$p < 0.001$; NS, not significant. CRC, colorectal cancer; qPCR, quantitative reverse transcription PCR.

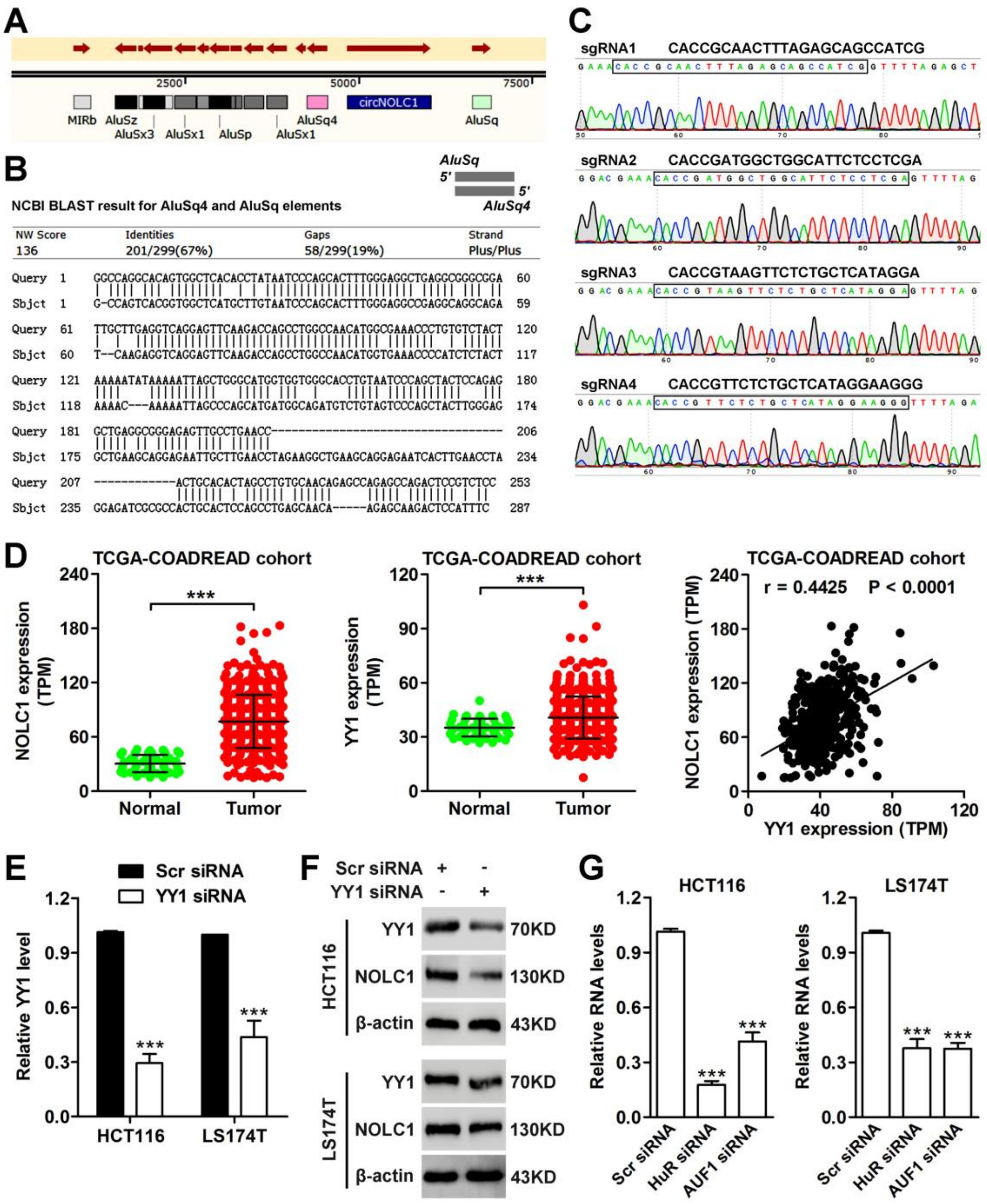

Figure S6. Related to Figure 6. (A) Schematics showing that the flanking introns of NOLC1 (exons 2-5) contained highly complementary Alu repeats with fourteen short interspersed elements in intron 1 and one short interspersed element (named AluSq) in intron 5. (B) The sequence of AluSq4 in the intron 1 was aligned to AluSq in the intron 5 of the NOLC1 gene using BLAST. Query, AluSq. Sbjct, AluSq4. (C) The four sgRNA expression plasmids were confirmed by Sanger sequencing. (D) The TPM values of NOLC1 (left) and YY1 (middle)

between CRC and normal tissues in the TCGA-COADREAD cohort. Correlation between NOLC1 and YY1 levels in CRC samples derived from TCGA (right). The Pearson correlation coefficient (r) with the significance is indicated. (E) Efficient knockdown of YY1 in CRC cells was detected by qPCR. (F) HCT116 and LS174T cells were transiently transfected with scramble or YY1 siRNA, and lysates were applied to western blot analysis. (G) Efficient knockdown of HuR or AUF1 in HCT116 and LS174T cells was detected by qPCR. ***$p < 0.001$. sgRNA, single-guide RNA; TPM, transcripts per million; CRC, colorectal cancer; TCGA, The Cancer Genome Atlas; siRNA, small interfering RNA; qPCR, quantitative reverse transcription PCR.

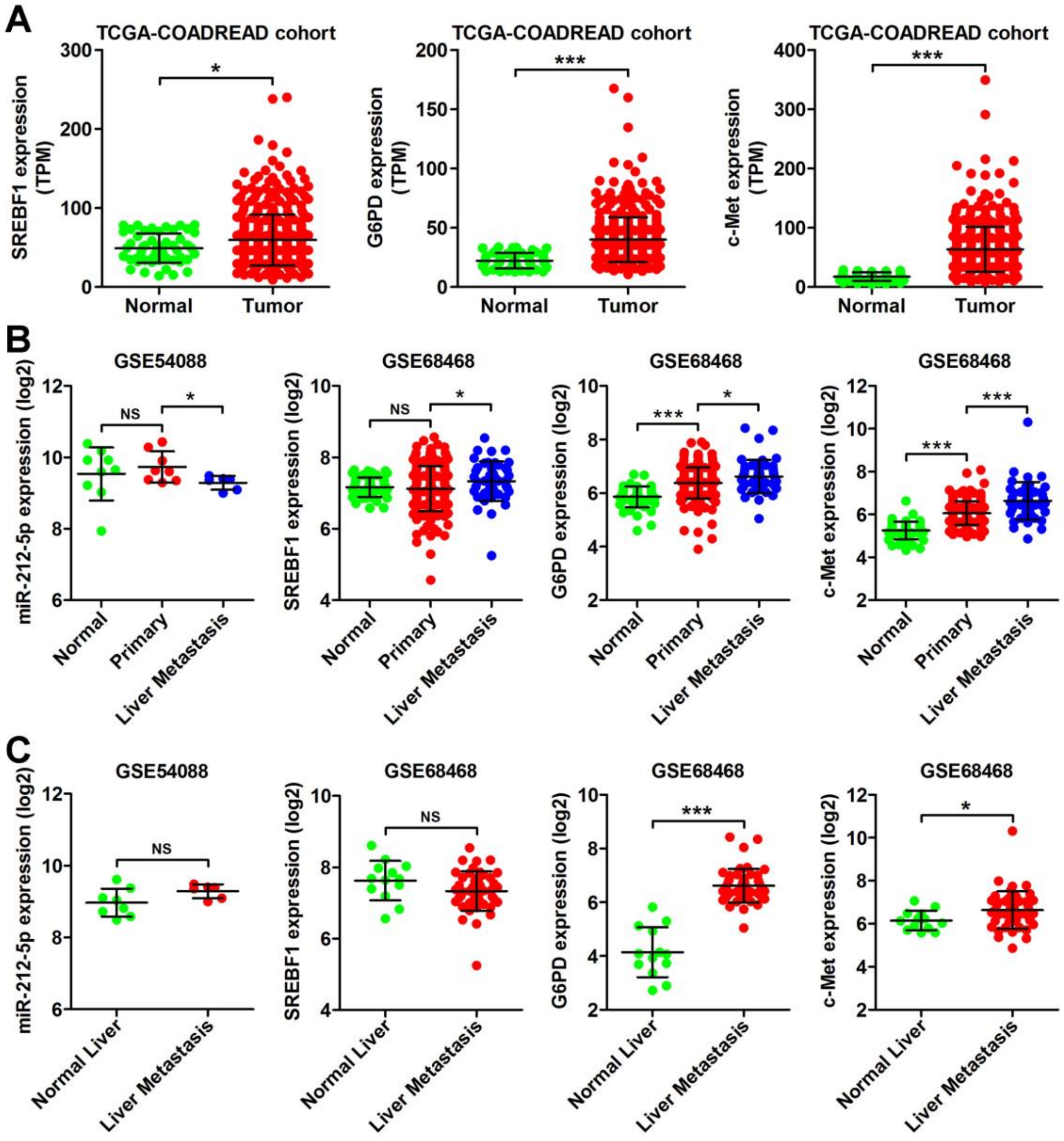

Figure S7. Related to Figure 7. (A) The TPM values of SREBF1, G6PD and c-Met in CRC and normal tissues in the TCGA-COADREAD cohort. (B) Analysis of the expression levels of miR-212-5p, SREBF1, G6PD and c-Met in normal colorectal mucosa, primary CRC, and liver metastasis samples in two GEO datasets (GSE54088 and GSE68468). (C) The expression levels of miR-212-5p, SREBF1, G6PD and c-Met in normal liver and liver metastasis tissues in GSE54088 and GSE68468. *p < 0.05, ***p < 0.001; NS, not significant. TPM, transcripts per million; TCGA, The Cancer Genome Atlas; CRC, colorectal cancer.
